# Role of *spt23* in *Saccharomyces cerevisiae* thermal tolerance

**DOI:** 10.1007/s00253-022-11920-3

**Published:** 2022-04-27

**Authors:** Zhilong Lu, Yanling Wu, Ying Chen, Xiaoling Chen, Renzhi Wu, Qi Lu, Dong Chen, Ribo Huang

**Affiliations:** 1 State Key Laboratory for Conservation and Utilization of Subtropical Agro-Bioresources, Guangxi University, Nanning, Guangxi 530004 People’s Republic of China; 2 College of Life Science and Technology, Guangxi University, Nanning, Guangxi 530004 People’s Republic of China; 3 National Engineering Research Center for Non-Food Biorefinery, Guangxi Academy of Sciences, Nanning, Guangxi 530007 People’s Republic of China

**Keywords:** *spt23*, Lipid acids, Transposon, Heat tolerance, Pathways

## Abstract

**Abstract:**

*spt23* plays multiple roles in the thermal tolerance of budding yeast. *spt23* regulates unsaturated lipid acid (ULA) content in the cell, which can then significantly affect cellular thermal tolerance. Being a Ty suppressor, *spt23* can also interact with transposons (Tys) that are contributors to yeast’s adaptive evolution. Nevertheless, few studies have investigated whether and how much *spt23* can exert its regulatory functions through transposons. In this study, expression quantitative trait loci (eQTL) analysis was conducted with thermal-tolerant *Saccharomyces cerevisiae* strains, and *spt23* was identified as one of the most important genes in mutants. *spt23*-overexpression (OE), deletion (Del), and integrative-expressed (IE) strains were constructed. Their heat tolerance, ethanol production, the expression level of key genes, and lipid acid contents in the cell membranes were measured. Furthermore, LTR (long terminal repeat)-amplicon sequencing was used to profile yeast transposon activities in the treatments. The results showed the Del type had a higher survival rate, biomass, and ethanol production, revealing negative correlations between *spt23* expression levels and thermal tolerance. Total unsaturated lipid acid (TULA) contents in cell membranes were lower in the Del type, indicating its negative association with *spt23* expression levels. The Del type resulted in the lower richness and higher evenness in LTR distributions, as well as higher transposon activities. The intersection of 3 gene sets and regression analysis revealed the relative weight of *spt23*’s direct and TY-induced influence is about 4:3. These results suggested a heat tolerance model in which *spt23* increases cell thermal tolerance through transcriptional regulation in addition to *spt23*-transposon triggered unknown responses.

**Key points:**

• *spt23 is a key gene for heat tolerance, important for LA contents but not vital.*

• *Deletion of spt23 decreases in yeast’s LTR richness but not in evenness.*

• *The relative weight of spt23’s direct and TY-induced influence is about 4:3.*

**Supplementary Information:**

The online version contains supplementary material available at 10.1007/s00253-022-11920-3.

## Introduction

Bioethanol is a renewable and sustainable energy source. However, environmental stresses such as high temperature, high osmosis, and toxic metabolites can inhibit ethanol fermentation by *Saccharomyces cerevisiae* (Bauer and Pretorius 2000). Consequently, yeast strains that are better adapted to these stresses are typically preferred for industrial purposes, and many attempts have been made to identify yeast strains with better tolerance to higher temperatures. Bioethanol industrial processes also benefit from heat-resistant yeast owing to the higher fermentation temperatures that are needed to: (1) promote higher catalytic activities that can theoretically produce higher bioethanol yields (Matsushita et al. 2016) and (2) reduce costs by lowering energy consumption in cooling and distillation systems (Hoshida and Akada 2017). These advantages become more prominent during high-gravity fermentation because yeast strains with high-temperature tolerances generally possess better osmotic tolerances. Higher temperatures are required for saccharification during simultaneous saccharification and fermentation, and these are better accommodated by thermal-tolerant yeast, thereby significantly reducing energy consumption. Many efforts have been consequently made to identify high temperature-tolerant yeast strains, including through physical/chemical mutagenesis, hybridization, and genetic engineering.

*spt23* is a potential genetic target for thermal-resistant yeast genetic engineering for two important reasons. First, Spt23p is a transcriptional regulator of *ole1* that encodes a Δ9 fatty acid desaturase and determines the unsaturated lipid acid (UFA) oleic oil content within cell membranes (Zhang et al. 1997, 1999). UFA content levels are highly related to the adaptation of yeast to various stresses, including high temperature, for example (Arthur and Watson 1976; Carratu et al. 1996; Chatterjee et al. 1997, 2000). *spt23* is also a chromatin-molding factor that is inferred to be a global transcriptional regulator (Dula and Holmes 2000; Rape et al. 2001). *spt23* may also be involved in thermal resistance by yeasts as a suppressor of Tys. Insertion of Ty1 upstream of *his4* induces a cold-sensitive *S. cerevisiae* phenotype (Burkett and Garfinkel 1994). Introducing *spt23* then inhibits transposon activity and alters the yeast’s thermal resistance capacity (Paquin and Williamson 1984). However, there has been little exploration of how *spt23* works within yeast thermal tolerance and whether/how transposons are affected by *spt23* during this process. Understanding how *spt23* functions in both pathways are thus important to better understand the heat shock response of yeast. Here, next-generation “omics techniques” (i.e., genome sequencing, RNA-seq, and LTR (long terminal repeats) amplicon sequencing), cell membrane fatty acid content evaluation, and qRT-PCR were used to identify the two pathways that *spt23* is involved in during heat shock stress.

An obstacle to assessing these mechanisms is the accurate determination and quantification of yeast transposon activity because Tys typically comprise repeat sequences that are interspersed in genomes and render it too complex for accurate genomic profiling (Fink et al. 1981; Garfinkel et al. 1985). Several methods have been used to overcome these problems, including phenotype-based, hybridization-based, amplification-based, and whole-genome sequencing techniques. All of these methods are useful in specific fields, but all have individual shortcomings (Fingerman et al. 2003; Winzeler et al. 2003; Singer and Burke 2003; Bergman and Quesneville 2007).

An LTR-amplicon sequencing method was used in this study to assess transposon activity during various treatments. The method features advantages for population diversity monitoring and is inexpensive and convenient. In the method, Tys and LTRs are amplified with specific primers to generate pools and are then sequenced with an NGS (next-generation sequencing) platform. LTR diversity dynamics are then evaluated to characterize transposon activities and inform population distribution analyses. Using these methods, we observed changed LTR alpha diversity in yeasts cultivated at 30 and 37 °C, suggesting the practicability of this method. After quantifying transposon activities, the level of *spt23* transcriptional regulation and transposon effects, is estimated quantitatively as approximately 4:3.

## Materials and methods

### Strains and plasmids

*S. cerevisiae* 1015 (*S. cerevisiae* CGMCC 2.4748) is an ethanol-yielding strain, and strain 101530 is a thermal-tolerant mutant, obtained through UV irradiation with 20 W of light power and a vertical distance dimension of 20 cm and then screening by grown in YPD medium at 45 ℃ for 72 h. The yeast expression vector pNC891 was kindly provided by Hackett et al. (2006). The gene-editing plasmid p415-GalL-Cas9-CYC1t for *S. cerevisiae* was kindly provided by DiCarlo et al. (2013). The plasmid pCrispr-con is derived from the p415-GalL-Cas9-CYC1t plasmid. A bacterial RFP (red fluorescent protein) cassette was placed in pCrispr-con for quick golden gate cloning, while a KanMX biomarker was used for easy positive selection of industrial yeasts using a constitutive promoter upstream of the Cas9 ORF (open reading frame) (Supplemental Fig. S1a). pPH4 is an over-expression plasmid for yeast (Supplemental Fig. S1b). The cloning vector pMD18 was purchased from Takara (Dalian, China), while the helper plasmid pMD-gp1 was constructed by inserting a gRNA-promoter inverted structure into the pMD18 plasmid (Supplemental Fig. S1c).

### Genomic resequencing of strains

DNA libraries were prepared with the Nextera DNA Library Preparation Kit (FC-121–1031, Illumina, San Diego, USA) following the manufacturer’s instructions, and genome sequencing was performed on an Illumina MiSeq platform using 300 bp paired-end sequencing. FastQC (v.0.11.7) was used for reads quality control, and Trimmomatic (v.0.38) was used to filter out low-quality reads and trim reads (Bolger et al. 2014). The BWA aligner (v.0.7.17) was used for reading and mapping, with *S. cerevisiae* R64-1 as the reference genome (Li and Durbin 2009). SNP (single-nucleotide polymorphism) calling was performed with GATK (v. 4.1.5.0) (McKenna et al. 2010).

### Transcriptomic sequencing

Strains 1015 and 101530 were cultivated in YPD medium at either 30 or 37 °C and sampled at 16 and 40 h, with 16 h corresponding to the mid-point of the logarithmic growth phase when yeast reproduces most rapidly and 40 h representing the stationary phase when ethanol accumulation peaks. Transcriptomic sequencing was performed at Novagene (Tianjin, China) on the Illumina NovaSeq sequencing platform. Read mapping and counting were conducted with HTSeq (1.99.2) (Anders et al. 2015), and the expression of different genes were evaluated with edgeR (2.1) (Robinson et al. 2010). Gene ontology (GO), Kyoto Encyclopedia of Genes and Genomes (KEGG) pathway, and Reactome pathway enrichment were evaluated with the KO-Based Annotation System (KOBAS) 3.0 program (Bu et al. 2021) and the Database for Annotation, Visualization and Integrated Discovery (DAVID) program (https://david.ncifcrf.gov/) (Dennis et al. 2003). eQTL analysis was conducted with PheNetic to infer mutants (De Maeyer et al. 2013, 2015). The genes harboring non-synonymous SNPs from genome sequences were used as source genes, overall expression data were considered the background, and significantly differentially expressed gene expression levels were used as weights for the analyses. PheNetic removes unimportant nodes while retaining drive mutations and their core interaction networks.

### Viability under stress conditions

To measure thermal tolerance, yeast strain aliquots were incubated in YPD at 45 °C, 50 °C, and 55 °C for 10 min, followed by plating, as with the other treatments. Strains grown in YPD at 30 °C were used as references. Three replicates were used for each sample. For other stresses, aliquots of yeast strains were exposed to 20% ethanol, 1% acetate, and 0.33% H_2_O_2_ for 10 min and then rinsed twice before plating on YPD solid medium and cultivated at 30 °C for 24 h. To induce caramel stress, strains were grown in YPD with 50% caramel at 30 °C for 1 h and then plated as in the other treatments. Colony counting was performed using the ImageJ software program (Grishagin 2015). Differences in characteristics between groups were analyzed using Kruskal–Wallis tests and pairwise Wilcoxon tests with Benjamini–Hochberg corrections.

### Construction of spt23 deletions, over-expression, and integral-expression recombinants

Two target sites in the 5′ direction of *spt23* were chosen for targeting, including 5′-CTGAAAATGATGAGTGGCAC-3′ and 5′-CTTGATCGACACGTTCAATT-3′. The insertion fragment was amplified using pMD-gp1 as the template. The fragment was then ligated to pCrispr-con through a one-pot golden gate reaction. Genomic DNA of transformants was extracted from a mixture of 20 colonies and then subjected to PCR verification of transformation success with the primers 5′-TTGGGCTAGCGGTAAAGGTG-3′ and 5′-TCGAAACGTGAGTCTTTTCCTTACC-3′. DsDecodeM (Xie et al. 2019) is a tool for rapid decoding of multiple superimposed sequencing chromatograms and was used for genotyping targeted mutations of *spt23*. To establish the overexpression strain (OE), wild type (WT) *spt23* was cloned into the pPH4 vector that is used for yeast overexpression (Supplemental Fig. S1b). An integrative expression strain that compliments the *spt23*Δ phenotype was also constructed (Supplemental Fig. S1d). A GFP-*spt23* (partial) cassette was synthesized with fusion PCR. The pADH1-GFP fragment was then amplified from the pNC891 vector. A fusion fragment with homologous recombinant arms to *spt23* was co-transformed with pCrispr-con-gRNA into *spt23*Δ strains. Transformants were then selected on a solid YPD medium with 300 mg/mL G418. Colonies were evaluated for GFP expression with fluorescence microscopy at an excitation wavelength of 438 nm. The recombinant strains were denoted as *spt23*::GFP-*spt23* or integrative-expressed (IE).

### Determination of lipid acids in cell membranes

Extraction of pure yeast cell membranes followed Panaretou and Piper (2006) but with modifications. Briefly, cell walls were enzymatically lysed with lyticase to obtain protoplasts. Protoplasts were then treated with SDS and heating in a microwave to release cell contents. Cell membranes were then separated from mitochondria and other organelles by super-high-speed sucrose gradient density centrifugation at 280,000 g at 4 °C for 14 h in a Beckman Optima L-90 K (Beckman Coulter, Nyon, Switzerland) ultracentrifuge. Cell membranes were picked with a sterile syringe at the inter-layer between 1.65 and 2.25 M. Cell membranes were then diluted and rinsed twice with Buffer A (2 mM EDTA and 25 mM imidazole, pH = 7). Lipid acid contents in cell membranes were measured using methods described by Folch et al. (1957). Detection of esterized lipid acids was conducted on a Thermo Trace1310 ISQ gas mass spectrometer (Thermo Fisher Scientific, MA, USA) equipped with a TG-5MS column.

### qRT-PCR

Ten genes involved in yeast transcriptional regulation, stress response, and lipid acid synthesis were selected for comparison of gene expression and verification of transcriptomic sequencing results. Among these genes, *mga2* is a homolog of *spt23*. *dog2* and *stf2* are indicators of yeast hyper-osmosis and ROS (reactive oxygen species) tolerance response initiation. *ole1*, *erg1*, and *acc1* are important factors involved in yeast lipid acid synthesis, and *rfu1* participates in ubiquitin homeostasis that is critical for proteasome functioning (Kimura et al. 2009; Wolf and Petroski 2009). The products of *stp3* and *swi5* are components of the SAGA and SWI/SNF complexes, respectively, which are both important regulons of RNA polymerase II (Koutelou et al. 2010; Sanz et al. 2016). Samples were collected after 16 h of culture at either 30 or 37 °C. Reverse transcription was achieved with a TaKaRa PrimeScript™ RT-PCR (TaKaRa, Tianjin, China) Kit, and qPCR was conducted with a SYBR® Premix Ex Taq™ II kit (TaKaRa, Tianjin, China) in an AnalytikJena qTOWERE2.2 instrument (AnalytikJena, Jena, Germany). qPCR reactions were conducted with three replicates, and relative expression was calculated with the 2^−ΔΔCt^ method. Expression at 16 h was used as the control, and expression of the *alg9* gene was used as the internal standard. qPCR primers are listed in Supplemental Table S1.

### LTR amplicon sequencing and transposon activity

**Fig. 1:**
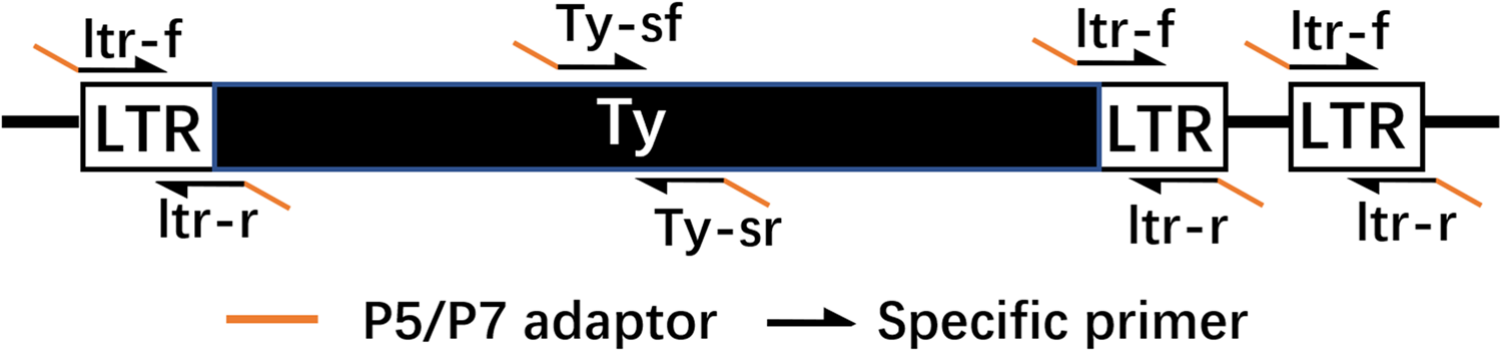
Ideogram of transposons, LTRs, and regions used for site-specific PCR amplification primers. ltr-f and ltr-r are the forward and reverse primers that amplify both complete transposon and obsolete LTR sequences

Specific primers were designed to amplify TY1-TY5 and their LTRs (Fig. 1). The LTR primers were designed to cover both the obsolete and complete LTR sequences and are shown in Supplemental Table S2. PCR products were pooled in equimolar levels to construct a library for sequencing on the Illumina MiSeq (Illumina, San Diego, USA) platform (300 bp, paired-end sequencing). Output sequences were joined with the FLASH program (1.2.11) (Magoč and Salzberg 2011) and annotated by aligning against *S. cerevisiae* Ty and LTRs sequences. Annotated sequences were then grouped and enumerated based on BLAST (Camacho et al. 2009) results. Pairwise sequence alignments and similarity matrices were constructed with ClustalW (Thompson et al. 2002). α-diversity and β-diversity levels of LTR sequence compositions were calculated with MOTHUR (1.45.0) (Chappidi et al. 2019; Schloss 2020). The 1015 WT grown at 30 °C was used as the reference, and transposon activity (Ta) was defined by LTR perturbation. Ta1 is generally defined as the mean of (LTR_n_/TY_n_)/(LTR_ref_/TY_ref_) for five transposon families (*n* = 1–5). Ta1 is copy number-sensitive and incapable of LTR sequence diversity profiling. Ta2 is typically calculated and also considers the diversity of LTR sequences, with calculation by (SI/TY)/(SI_ref_/TY_ref_), where SI is the Shannon index value for LTRs in a given sample.

### Weighting of spt23-affected pathways in thermal tolerance

A set of genes that were differentially expressed independently of strain and growth phase were identified and referred to as the H-CORE gene set. Genes from the PheNetic inferred core interaction networks were denoted as M-CORE and represented *spt23*-affected genes during thermal tolerance adaptation. Another set of genes comprising the first out-going neighbors of *spt23*-affected transposons was denoted as the T-CORE gene set. Thus, the intersecting genes of the H-CORE and M-CORE sets reflect the weight of *spt23*’s role in thermal tolerance. In addition, the intersecting genes of the H-CORE and T-CORE sets reflect the weight of transposon effects in thermal tolerance. From the perspective of influence weights, TULAs (total unsaturated lipid acids) and transposon activity were considered variables representing direct regulation by *spt23* and indirect regulation by transposons during yeast thermal tolerance. A regression model was then built where the partial correlation coefficients reflected the importance of variables.

## Results

### Construction of an *spt23* knock-out in addition to over-expression and integral-expression strains

The *spt23*Δ recombinant strain was constructed and confirmed with PCR using degenerate sequence analysis and Sanger sequencing (Supplemental Fig. S2). The *spt23* over-expression recombinant was subsequently verified by PCR. The integral-expression recombinant strain was also verified by PCR and GFP expression examination using fluorescence microscopy (Supplemental Fig. S3).

### Growth and ethanol fermentation

**Fig. 2:**
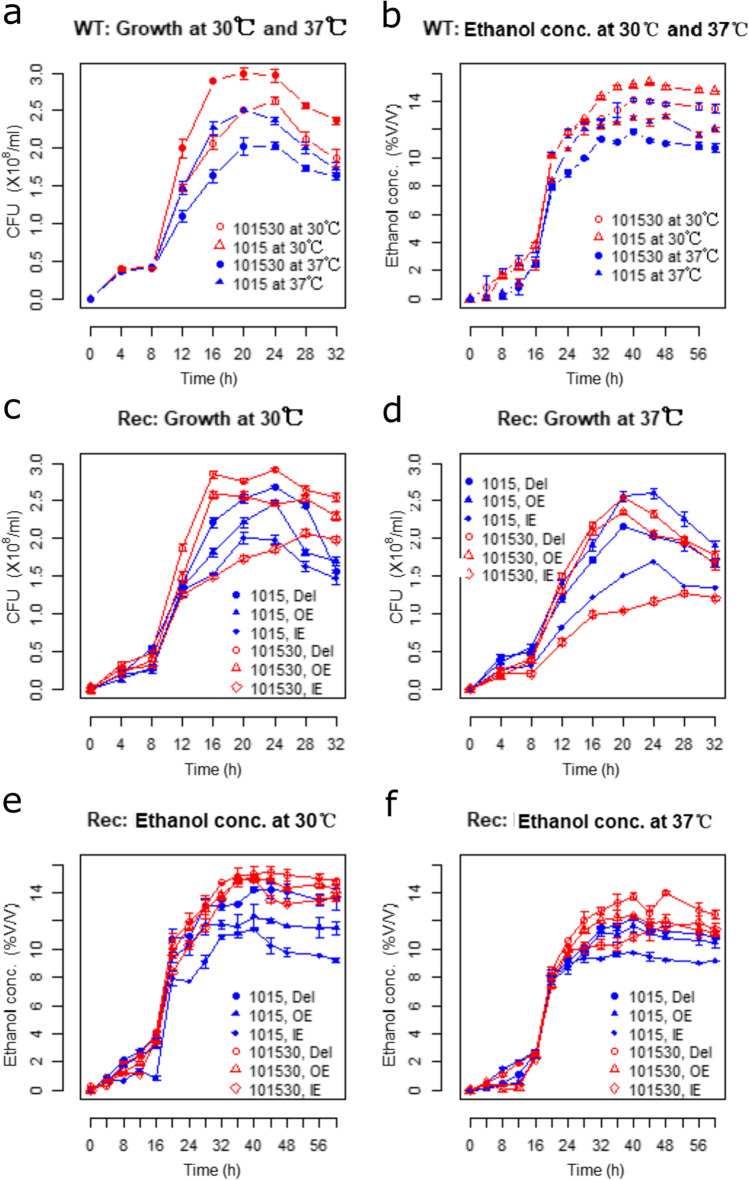
Growth curves and ethanol fermentation activities of *spt23* recombinant strains when grown at 30 or 37 °C

*S. cerevisiae* strain 101530 exhibited a higher growth rate and accumulated biomass than strain 1015 at either 30 or 37 °C (Fig. 2a). The highest level of biomass (2.95 × 10^8^ and 2.55 × 10^8^ CFU (colony forming unit)) was achieved at 24 h, representing 18 and 28.8% higher levels than that of strain 1015 at 30 or 37 °C. The growth of strains 101530 and 1015 at 30 °C was faster than at 37 °C. Better growth performance for strain 101530 was observed at 37 °C. Recombinant 101530 strains (Del, OE, and IE) exhibited higher biomass accumulation than 1015 (+ 0.02 × 10^8^ − 0.1 × 10^8^) at 30 °C. Biomass peaks for strain 101530 types Del and OE were observed at 0.01 × 10^8^ CFU/mL when grown at 37 °C, representing 0.27 × 10^8^ CFU/mL higher values than biomass peaks for strain 1015. In contrast, the IE type exhibited lower peak biomass than strain 1015 (− 0.51 × 10^8^). Accumulated biomass generally followed the sequence of Del > WT > OE > IE for both strains 101530 and 1015 (Fig. 2c, d).

Peak ethanol production concentrations for strain 101530 at 30 and 37 °C were 15.33 + 0.06% (*V/V*) and 12.77 + 0.2% (*V/V*), respectively, and for strain 1015 were 14.13 + 0.12% (*V/V*) and 11.87 + 0.19% (*V/V*), respectively (Fig. 2b). Recombinants of 101530 exhibited cultures with higher ethanol concentration than those of 1015. Ethanol concentrations of the WT and recombinant cultures followed the same trends as their growth, with the Del type exhibiting the highest ethanol yield of 15.47 ± 0.53% (*V/V*) at 30 °C and 14.0 ± 0.14% (*V/V*) when grown at 37 °C (Fig. 2e, f).

### Stress resistance

**Fig. 3:**
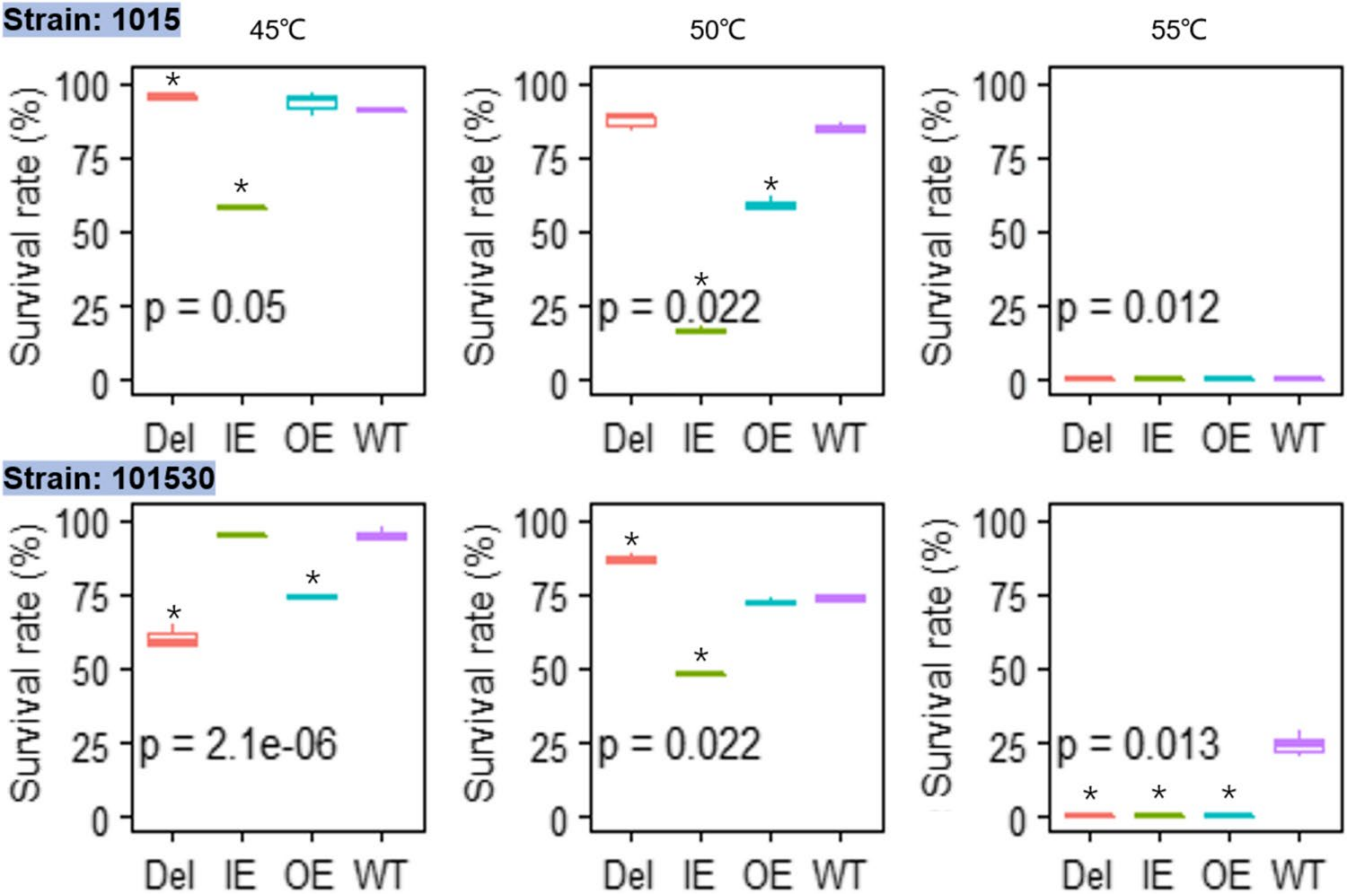
Survival rate of *spt23* recombinant strains after high temperature shocking

Heat resistance was evaluated by survival rate. The WT strain 101530 had the best heat tolerance capacity, especially at 55 °C, with a survival rate of 25% (compared to 0% for the other strains). Significant differences were observed in survival among recombinant types at 45 and 50 °C for both strains 101530 (*p* = 0.0051 and *p* = 0.0041, respectively) and 1015 (*p* = 00,097 and *p* = 0.0037, respectively), in addition to 55 °C for 101530 (*p* = 0.0022). The Del types of strains 101530 and 1015 exhibited lower survival than the WT at 45 °C, 50 °C, and 55 °C, with the exception of strain 101530 at 50 °C. The OE and IE types exhibited lesser heat tolerance than the WT at all times (Fig. 3).

The survival rate significantly increased for the Del type when grown on 20% ethanol YPD plates but decreased for the OE type. No significant increase was found for any type of strain 101530 when grown with 1% acetate, but a 71.6% increase in growth was observed for the deletion type of 1015. A Kruskal–Wallis test indicated a significant difference in growth due to 0.33% hydrogen peroxide stress, while a post hoc test (Dunn’s test with a Benjamini adjustment) did not support the presence of a valid difference between the WT and other recombinants for strain 101530. The survival rate of the over-expression type decreased by 35.44% because it was incapable of growing under 0.33% hydrogen peroxide stress. During 50% caramel stress, only the over-expression 101530 type exhibited a significant increase in caramel tolerance (Supplemental Fig. S4).

### SNPs, expression differences, and key drive mutations

The total number of SNPs and indels observed for the 101530 strains were 12,605 and 362, respectively, which were less than that observed for strain 1015 (Supplemental Tables S3 and S4). A total of 1900 genes were identified with sense mutations from SNPs and indels, making them important for drive mutation analysis. The total number of differentially expressed genes is shown in Table 1. Differentially expressed genes between 30 and 37 °C grown strains were subjected to KEGG and Reactome pathway enrichment analysis. At 16 h, strain 101530 expression was enriched in the ribosome (sce03010) and metabolic pathways (sce01100), while strain 1015 expression was only enriched with ribosome pathways (Supplemental Tables S5 and S6). Reactome pathway translation-associated terms (R-SCE-72737, 72613, and 72766) were enriched in strain 1015, and RNA-processing-associated terms (R-SCE-8953854, 6791226, 8868773, 72312) were enriched for strain 101530. At 40 h, neither KEGG nor Reactome pathway expression was enriched for strain 101530, while the expression of three RNA-processing-associated terms (R-SCE-6791226, 8868773, and 72312) was enriched in strain 1015 (Supplemental Table S7).

**Table 1:** Differentially expressed genes when comparing strains 101530 and 1015 grown at 30 and 37 °C with samples taken at 16 and 40 h

Strain	37 vs 30 °C	Number of genes	
		16 h	40 h
1015	Downregulated	752	734
	Upregulated	614	693
101530	Downregulated	977	1043
	Upregulated	956	1054

Intersection analysis of the four-gene set groups in Table 1 yielded the identification of the H-CORE gene set, representing a thermal resistance-related core gene set independent of strain and growth phase (Supplemental Table S8). GO enrichment analysis of the 258 H-CORE genes indicated that they were enriched in ribosome synthesis functions (especially rRNA processing) and protein targeting/translation functions (Table 2).

**Table 2:** GO enrichment analysis of H-CORE genes

GO term		GO-ID	N*	B*	p*
MF^a^	-	-			-
CC^b^	Mitochondrion	5739	77	1189	2.16E-04
	Intracellular ribonucleoprotein complex	30,529	28	309	0.004
	Nucleolus	5730	25	271	0.006
	Mitochondrial matrix	5759	15	128	0.015
	Pwp2p-containing subcomplex of 90S preribosome	34,388	4	6	0.032
BP^c^	Mitochondrial translation	32,543	19	120	2.94E-04
	Ribosome biogenesis	42,254	21	199	0.017
	Endonucleolytic cleavage in 5′-ETS of tricistronic rRNA transcript (SSU-rRNA, 5.8S rRNA, LSU-rRNA)	480	8	31	0.017
	Protein targeting to mitochondrion	6626	11	65	0.017
	Endonucleolytic cleavage to generate mature 5′-end of SSU-rRNA from (SSU-rRNA, 5.8S rRNA, LSU-rRNA)	472	8	31	0.017
	rRNA methylation	31,167	10	59	0.032

^a^Molecular function; ^b^Cellular component; ^c^Biological process; N*, input number; B*, background number; p*, corrected *p*-value

Genes with sense mutations and genes exhibiting differential expression were used to drive mutation tracking with the PheNetic program. The networks affected by drive mutations are shown in Figs. 8 and 9, while the primary drive mutations inferred by PheNetic are listed in Supplemental Table S9. *spt23* was considered the 1st (at 16 h) and 2nd (at 40 h) most important influencer of the transcriptomes. Genes in the network were extracted and denoted as M-CORE (Supplemental Table S10).

### Expression of key genes

**Fig. 4:**
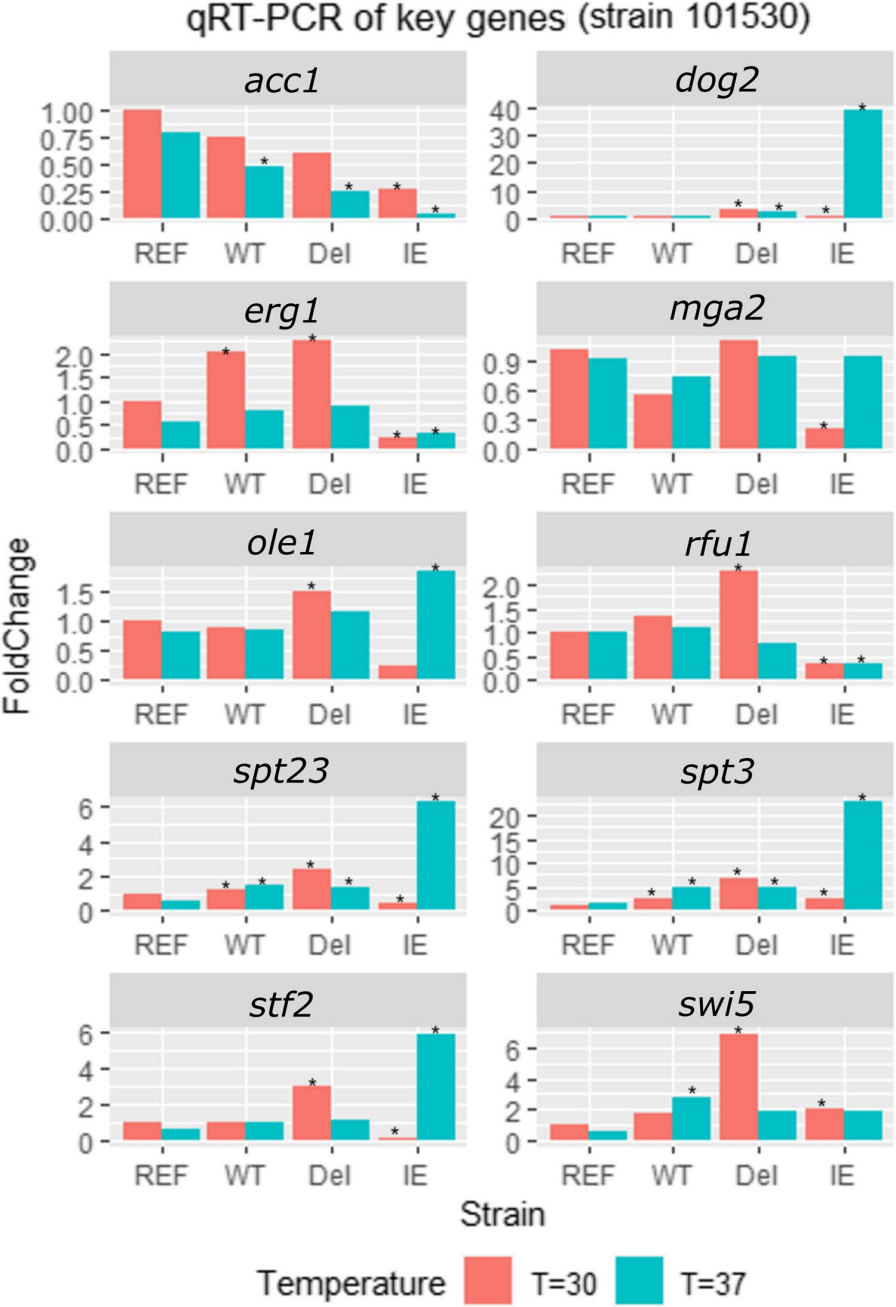
Gene expression of 10 key genes of *spt23* recombinant strains based on real-time fluorescence quantitative PCR. The reference sample for expression comparison is the 1015 wild-type strain grown at 30 °C. “^*^” is the indicator of significant difference to the reference

*spt23* expression levels were six-fold higher (based on FC) when the IE type was treated with heat. The *dog2*, *ole1*, *spt3*, *mga2*, and *stf2* expression levels exhibited similar increases in the IE type. Deletion of *spt23* caused a sharp decrease in *acc1*, *erg1*, *rfu1*, *stf2*, and *swi5* expression levels when yeast was exposed to heat treatment (Fig. 4). At 30 °C, the expression levels of *dog2*, *stf2*, *rfu1*, *erg1*, *ole1*, and *swi5* were significantly and positively correlated with *spt23* expression (*R*^2^ > 0.7) (Supplemental Fig. S7a). At 37 °C, the expression levels of *dog2*, *stf2*, and *ole1* exhibited positive correlations with *spt23* expression levels (*R*^2^ > 0.7), while *rfu1* levels were negatively correlated with *spt23* levels (*R*^2^ > 0.7) (Supplemental Fig. S7b).

### SLA contents in cell membranes

Saturated lipid acid (SLA) contents increased (by + 1.8 and 18.3% for strains 1015 and 101530) when WT strains were cultured at 37 °C relative to levels when grown at 30 °C. The Del type exhibited the same SLA trend increase as the WT (+ 2.8%) due to increased palm oleic acid and decreased oleic oil contents. Although all the WT and Del types exhibited lower SLA contents when grown at 37 °C, varying compositional changes were observed. WT strain 101530 exhibited an increase in SLA by decreasing longer chain lipid acid contents (e.g., palmitic and arachidonic acids), while WT strain 1015 primarily exhibited decreased short-chain lipid acids (e.g., pentadecanoenoic acid). In contrast, the IE type exhibited decreased SLA contents (− 7.2%) (Supplemental Table S11). A weak correlation was observed between *spt23* expression level and ULA (Unsaturated lipid acids) contents, indicating a non-dominating influence of *spt23* (*R*^2^ = 0.3) (Supplemental Fig. S8).

### LTR diversity and transposon activity

Differences in LTR alpha diversity metric values (i.e., Shannon evenness) between 30 and 37 °C grown cultures were significant (*p* = 0.043), while significant decreases were observed for OTUs (*p* = 0.029) that represented clustered groups of LTRs. Significant differences were not detected for the Shannon diversity index values or Chao richness estimations (Table 3 and Supplemental S12). Thus, LTR diversity was altered when *S. cerevisiae* was cultured under heat stress, especially when considering LTR evenness and overall OTU counts. However, correlations between transposon activities (Ta1 and Ta2) with *spt23* expression levels were not significant (Supplemental Fig. S9).

**Table 3:** Changes in different alpha-diversity metrics when comparing strains grown at 30 vs 37 °C

Alpha-diversity	Levene’s test	Shapiro–Wilk test	Significance
Shannon^a^	0.894	0.878	0.43
Chao^b^	0.001^c^	0.079	1.00
Shannon Evenness^a^	0.286	0.604	0.043^*^
OTU^a^	0.971	0.593	0.029^*^

^a^Single tailed *t*-test; ^b^Mann-Whitney *U*-test; ^c^Non-normal distribution; ^*^p < 0.05

### Weighing *spt23*’s role in thermal tolerance

**Fig. 5:**
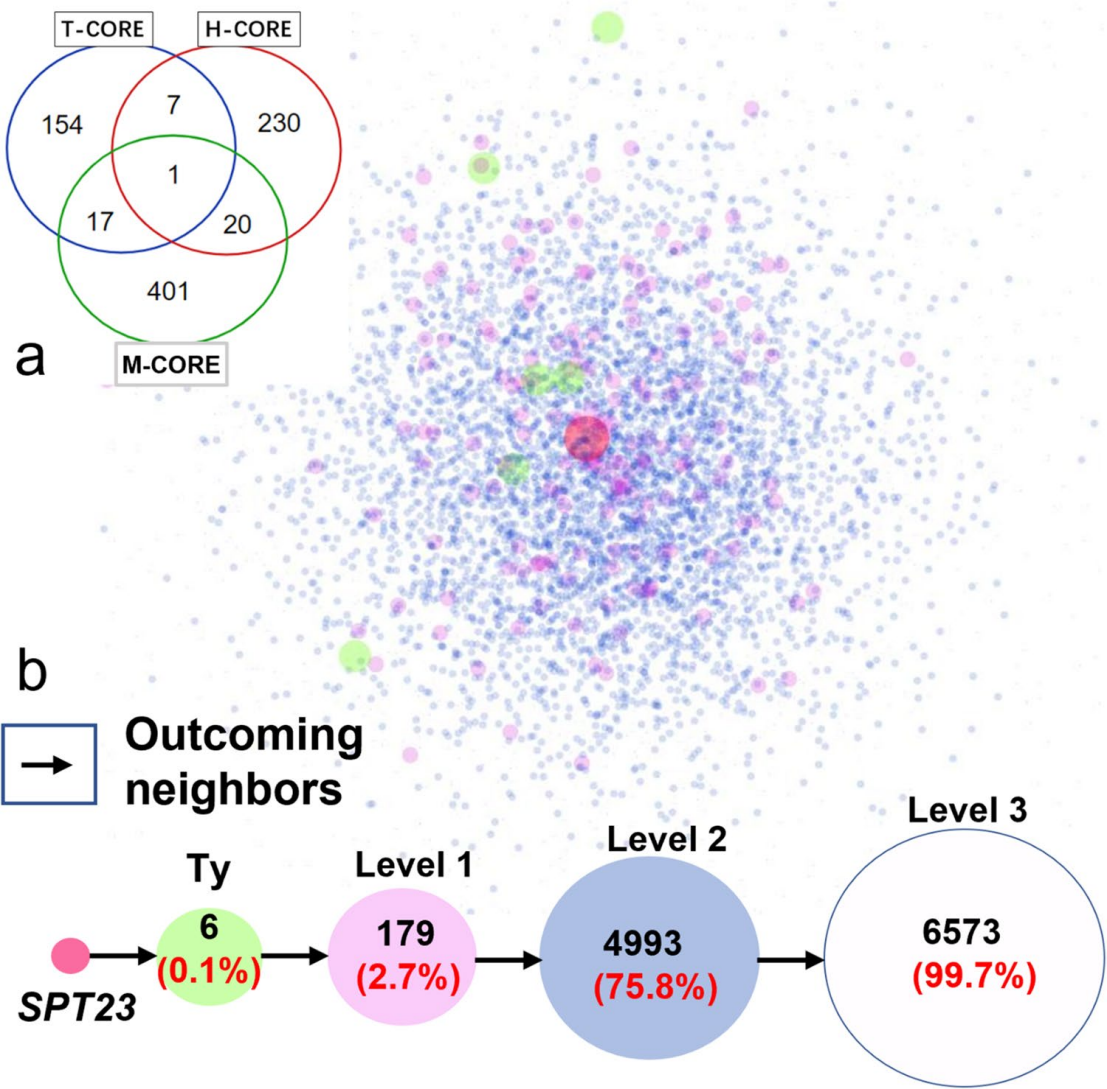
Interaction network of *spt23*-TY and the intersection of H-CORE, M-CORE, and T-CORE gene sets. **a** Intersection of H-CORE, M-CORE, and T-CORE gene sets. **b** T-CORE is yielded at level 1 of *spt23*-Ty’s outcoming neighbors

To evaluate how *spt23* regulates the heat-shock response through different mechanisms, the *spt23*-mutation-driven core network genes (M-CORE) and *spt23*-transposon-regulated core genes (T-CORE, Supplemental Table S13) were extracted and compared with the H-CORE gene set (Fig. 5). When considering overlapping genes, the ratio of direct contribution of *spt23* to high-temperature tolerance and indirect contribution through transposons was 1.31 (9.13:6.98%).

A regression model of survival rate to TULA contents and transposon activity (Ta2) was statistically significant (*p* = 0.015, Table 4), while a multi-correlation coefficient *R* (0.903) indicated a strong regression relationship. The adjusted *R*^2^ = 0.741 represents the total explanatory power of the two variables for the total variance and represents the combined direct and indirect influences of *spt23*. The regression coefficients of TULA and Ta2 exhibited *p* = 0.005 and *p* = 0.043, and thus neither factor should be excluded in the model. The partial correlation coefficients for TULA and Ta2 were − 0.90 and 0.77, respectively. Weights were evaluated with squared partial correlation coefficients, revealing that TULA and Ta2 explain 0.810 and 0.591 of the total residuals, respectively (Table 5). The weight of the *spt23* functions through a transcriptional regulatory role and Ty caused regulation was 1.37 (0.810:0.591). Thus, TULA was the most important factor conferring thermal tolerance, while transposons exhibited lower influences. The regression model was established as Survival45 =  − 2.39⋅TULA + 69.98⋅Ta2 + 152.43.

**Table 4:** Summary of the regression model survival rate (TULA^*^, Ta2^**^)

*R*	*R*^2^	Adjusted *R*^2^	Std. error	Change statistics		
				*R*^2^	*F*	Sig. F
0.903	0.815	0.741	9.18875	0.815	11.014	0.015

**Table 5:** Coefficients of regression for the regression model survival rate

Model		Unstandardized coefficients		Standardized coefficients	*t*	Sig	Correlations			Collinearity statistics	
		*B*	Std. error	Beta			Zero-order	Partial	Part	Tolerance	VIF
	Const	152.43	22.80		6.686	0.001	-	-	-	-	-
	TULA	− 2.39	0.51	− 1.06	− 4.69	0.005	− 0.739	− 0.90	− 0.90	0.72	1.39
	Ta2	69.98	25.98	0.61	2.69	0.043	0.048	0.77	0.52	0.72	1.39

^*^Total unsaturated lipid acids; ^**^Transposon activity evaluated by LTR diversity change

## Discussion

### Ribosome pathways are highly associated with thermal tolerance

Analysis of differentially expressed genes suggested that high-temperature tolerance mechanisms were associated with ribosomal synthesis and several metabolic pathways. The ribosome synthesis pathway plays important role in *S. cerevisiae*’s heat-shock response. Regulation of ribosomal genesis involves over 100 regulons that are primarily associated with RNA polymerase II (Wade et al. 2006). Sakaki et al. (2003) investigated heat-stressed yeast cell responses, observing that 122 ribosome-associated genes were downregulated alongside the upregulation of glycolysis. This could be attributed to the accumulation of damaged proteins after heat shock. Reductions in ribosomal protein expression have been observed in many studies and are thought to result from transcriptional arrest (Mager and Ferreira 1993). When adapting to stressful conditions, proteosomes strengthen the curation and degradation of damaged proteins. In addition, pools of newly synthesized ribosomes are required for normal cell functioning under stressful conditions. A whole-genome study by Auld et al. (2006) revealed that Spt23p binds 235 gene cassettes, including those for proteosomes in addition to ribosome- and glycolysis-associated genes. Grigull et al. (2004) validated the hypothesis for turbulence in ribosomal biogenesis in transcriptional regulation levels using heat shock through microarrays. Other studies have indicated a semi-housekeeping role played by *spt23* (in addition to its homologous counterpart) in lipid acid metabolism along with proteosomes (Wingender et al. 2001). Proteosomes and ribosomes are key contributors to protein homeostasis, and their close interactive relationships with *spt23* may provide clues for *spt23*’s role in *S. cerevisiae*’s heat shock response (Raskin et al. 2012).

Yeast’s thermal tolerance is associated with transcription factors that are widely distributed in cells and are strongly influenced by iron ion transport (Table 2). GO term enrichment analysis of H-CORE revealed key roles for iron ion transport in the response. Iron ion transport participates in metal ion homeostasis during exposure to environmental stresses (Wingender et al. 2001). Iron ions are critical for heme and sulfur-ferroproteins and are involved in energy generation via cellular respiratory functions (Raskin et al. 2012). Furthermore, metal ions are critical co-factors involved in cellular redox balance and thus critically contribute to stress tolerance through these pathways.

### Tolerance to different environmental stresses

Stress responses are complicated and intertwined (Bauer and Pretorius 2000). Cellular pathways associated with stress response respond to various environmental stresses, like the stress response element (STRE) signaling pathways for example (Ruiz-Roig et al. 2012). Many studies have highlighted the close relationships between other stress responses (Piper 1995; Watson and Cavicchioli 1983; Jimoh et al. 2013; Moraitis and Curran 2004, 2007; Wang et al. 2012; Kim et al. 2006). In this study, stress manipulations of *S. cerevisiae spt23* led to strain-dependent effects. The strain 1015 Del type exhibited higher ethanol tolerance than the WT 1015 strain, while the strain 101530 integral expression type exhibited higher ethanol tolerance than WT 101530. Deletion of *spt23* increased acetate tolerance by strain 1015, but similar significant changes were not detected for any genotype of strain 101530. Likewise, tolerance to hydrogen peroxide increased in strain 1015 by over-expression of *spt23*, but similar significant differences were not observed for the 101530 strains. While the over-expression type of strain 101530 exhibited higher caramel tolerance, manipulation of *spt23* altered the caramel tolerance of strain 1015. These results contrast with the expectation that tolerance to all stresses should be similar for all strains or across a specific manipulation. Rather, differences in phenotype between strains 1015 and 101530 were more prominent than differences due to treatment. Nevertheless, the results also suggest that *spt23* does not dominate yeast stress tolerance responses alone as expected. In contrast, the stress tolerance phenotype represents the complex coordination of multiple genes.

### Profiling *spt23*’s role in transcription regulation

*mga2* is a homolog of *spt23*, and deletion of either gene generates mild suppression of yeast growth, while simultaneous loss of the two genes is lethal to yeast (Zhang et al. 1997). Both genes are involved in *ole1* transcriptional regulation (Zhang et al. 1999). However, *mga2* expression is more sensitive to ULA feedback repression than *spt23* expression (Hoppe et al. 2000). In addition, *mga2* strongly influences *ole1* under oxygen-limited conditions, while *spt23* does not (Jiang et al. 2001). Synergism of the two genes is critical to ULA content homeostasis in yeast cells, but *spt23* apparently only plays an auxiliary role. However, *spt23* exhibits a wider interaction spectrum across the whole genome. A whole-genome study using microarrays revealed that 235 genes are bound to Spt23p (Auld and Silver 2006). In this study, *mga2* expression levels were consistent among different types, and no significant correlation was found between *spt23-mga2* expression levels.

Firstly, transcriptional regulation of *spt23* was first evaluated with *spt3* and *swi5*. The expression levels of *spt3* significantly increased when grown at 37 °C, and a high correlation between *spt23* and *spt3* expression was observed. This result suggests that the SAGA complex may be involved in the yeast heat shock response. However, it remains unclear whether *spt23* is a regulator of *spt3*, or if both are regulated by other factors. A 71.4% decrease in *swi5* expression levels indicated that the SWI/SNF complex was absent in the regulation of the RNA polymerase II dominated stress response of *S. cerevisiae*.

Secondly, lipid acid content regulation of *spt23* was evaluated due to influences of *ole1*, *erg1*, and *acc1*. *ole1* is a direct subject of *spt23* regulation. Significantly increased expression levels when grown at 37 °C resulted from *spt23* over-expression in the IE type. Previous studies have revealed the contribution of ergosterol to tolerating environmental stresses (Yoshioka and Hashimoto 1983). Ergosterol and other long-chain saturated lipid acids are considered enhancing factors for heat shock resistance (Swan et al. 1998). In this study, *erg1* did not yield a positive effect in thermal tolerance augment. *acc1* is the initial enzyme for yeast lipid acid synthesis, and its expression was decreased in the heat treatment for all genotypes, thereby clearly indicating it is a subject of *spt23* regulation.

Thirdly, synergistic effects from different stresses on the regulation of *spt23* were evaluated with *dog2* and *stf2*. The expression levels of *dog2* and *stf2* drastically increased after temperature shifts. Furthermore, the effects of phenotype on stress tolerance to both caramel and H_2_O_2_ were strain-dependent. Thus, the regulation of *spt23* is highly complicated, and additional investigations are needed to assess the mutual effects of these genes.

Finally, significant differences were not observed in the expression level of *rfu1*, indicating few influences of *spt23* on ubiquitin homeostasis.

### Lipid acid compositions altered by *spt23* modification

Cellular lipid composition influences stress activation through the STRE (stress response element) mechanism (Chatterjee et al. 2000). Cellular membrane fatty acid compositional measurements revealed differences in fatty acid contents for different *spt23*-modified strains. Generally, saturated fatty acid content increases when growth temperatures shift from 30 to 37 °C. The survival rates at 45 °C, 50 °C, and 55 °C revealed that the contents of unsaturated fatty acids and high-temperature tolerance were negatively correlated. Similar results were observed by Kimura et al. (2009). The qPCR results indicated that the contents of unsaturated fatty acids were positively and weakly correlated with *spt23* expression. Zhang et al. (1999) reported similar conclusions. The weak correlation may result from *mga2*, which exerts a complementary role to *spt23* in lipid acid desaturation. Strain 101530 exhibited a higher survival rate after 50 °C heat shock, while strain 1015 strain exhibited a higher survival rate after 45 and 55 °C heat shock. This difference may be attributable to different transitions of cell membrane fatty acids, wherein strain 101530 mainly relies on reduced palmitoleic and arachidonic acid, while strain 1015 strain reduces membrane levels of pentadecanoic acid. The overall influence of *spt23* on non-saturated fatty acid regulation is limited and not necessarily a determinant of the stress response, although it is nevertheless substantially involved.

### Weaker influence from the Ty regulatory pathway compared to transcriptional regulation

LTR sequencing was used to evaluate the regulatory roles of specific genes on transposons. Perturbation of *S. cerevisiae* transposon LTR diversity was then observed after heat shock. The α-diversity values of LTRs, including Shannon index values, Chao richness estimation values, and OTU numbers, all decreased for Del-type strains after shifts in conditions, while these values increased for IE-type strains. Furthermore, Shannon evenness indices significantly increased, indicating higher LTR population evenness. The *spt23* dosage levels apparently resulted in less LTR population diversity but more evenness in LTR distributions after heat treatment.

A contradictory result is the high level of correlation between *spt23* expression level and transposon activities (Ta). This result implies that transposon activities are highly associated with temperature shifts (i.e., environmental stresses) aside from *spt23* expression levels. If transposon activity was independent of *spt23* regulation, it should be an intrinsic characteristic of yeast strains. It is also possible that transposons are regulated by other genes or co-regulated with *spt23* under various stresses. Nevertheless, further studies are needed to verify this hypothesis since the sample count is inadequate for rigorously evaluating it.

TE-induced environmental adaptions have been observed in the fission-dividing yeast *Schizosaccharomyces pombe* (Esnault et al. 2019), wherein transposable element locations were strongly associated with numerous stress response genes. Similar transcription activation via promoter attenuation or replacement by transposons has also been observed in *S. cerevisiae* (Servant et al. 2008). Indeed, transposon-induced gene copy number variation within genomes can promote yeast adaptive evolution (Infante et al. 2003; Mourier and Willerslev 2010). These observations suggest that the potential for transposon involvement in adaptation should not be underestimated. Additional analysis of yeast interactive regulatory networks would help to illuminate the scope of transposon-influenced regulation.

Both gene numbers and influential weight between *spt23*-direct and *spt23*-transposon pathways were roughly ~ 4:3, indicating weaker influences from transcription regulation and transposon effects.

### A heat tolerance model with *spt23* and transposons

**Fig. 6:**
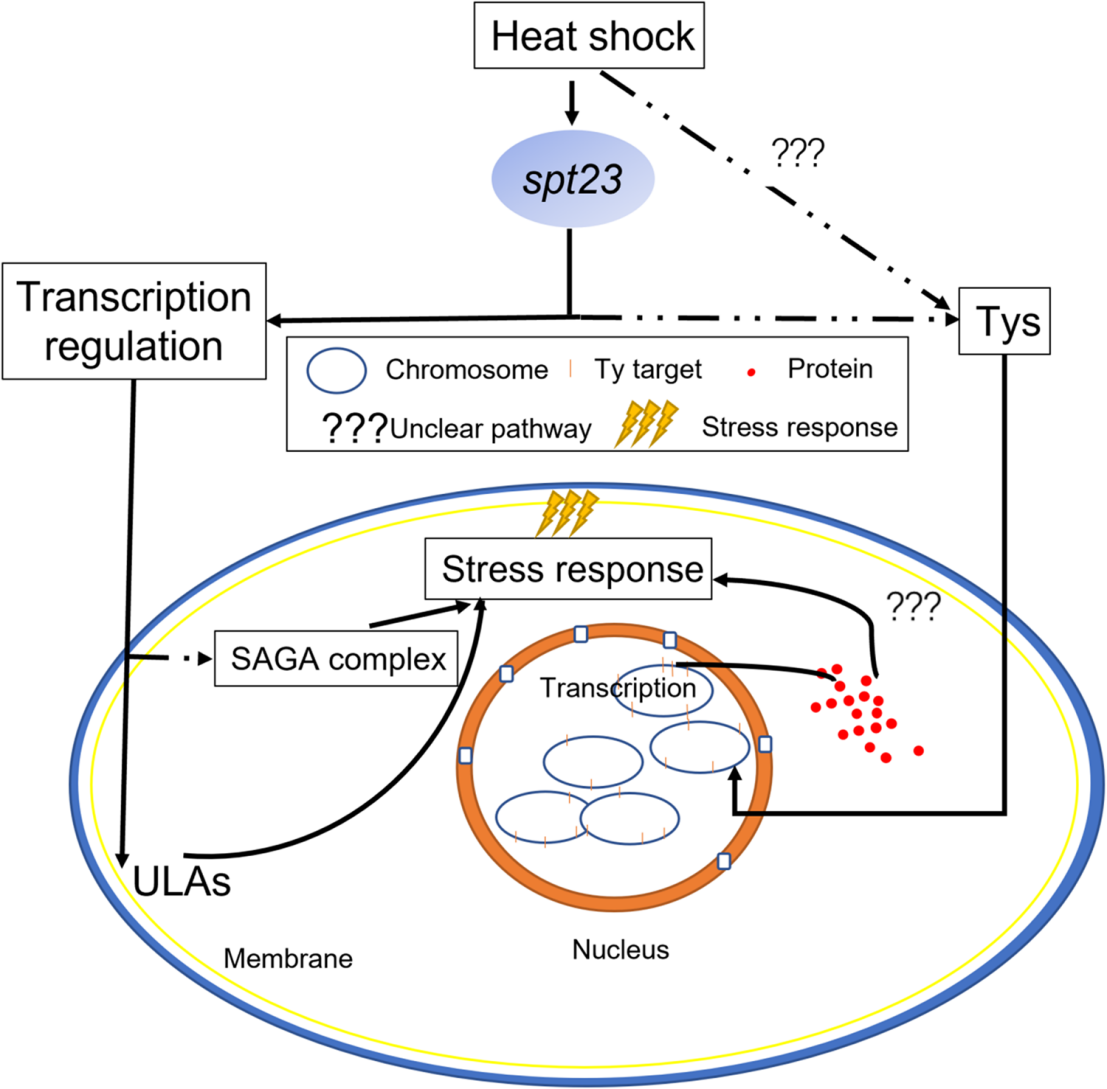
Schematic demonstrating how *spt23* is involved the *Saccharomyces cerevisiae* heat tolerance response

A novel model was established based on the transposon influence of yeast thermal tolerance (Fig. 6). In the model, *spt23* increases cell thermal tolerance by transcriptional regulation of the SAGA complex, while *ole1* regulation modifies lipid acid contents in the cell membrane. In addition, the interaction of *spt23* and transposons, or transposons by themselves, trigger unknown regulatory pathways that confer thermal tolerance to yeast cells. The relative weights of the two mechanisms were estimated as 1.31:1 ~ 1.37:1, or approximately 4:3.

## Supplementary Information

Below is the link to the electronic supplementary material.

Supplementary file1 (PDF 2630 KB)

## Data Availability

The NGS sequences were uploaded to NCBI RSA under BioProject ID PRJNA799192.

## References

[CR1] Anders S, Pyl PT, Huber W (2015). HTSeq–a Python framework to work with high-throughput sequencing data. Bioinformatics.

[CR2] Arthur H, Watson K (1976). Thermal adaptation in yeast: growth temperatures, membrane lipid, and cytochrome composition of psychrophilic, mesophilic, and thermophilic yeasts. J Bacteriol.

[CR3] Auld KL, Silver PA (2006). Transcriptional regulation by the proteasome as a mechanism for cellular protein. Cell Cycle.

[CR4] Auld KL, Brown CR, Casolari JM, Komili S, Silver PA (2006). Genomic association of the proteasome demonstrates overlapping gene regulatory activity with transcription factor substrates. Mol Cell.

[CR5] Bauer FF, Pretorius IS (2000). Yeast stress response and fermentation efficiency: how to survive the making of wine - a review. S Afr J Enol Vitic.

[CR6] Bergman CM, Quesneville H (2007). Discovering and detecting transposable elements in genome sequences. Brief Bioinform.

[CR7] Bolger AM, Lohse M, Usadel B (2014). Trimmomatic: a flexible trimmer for Illumina sequence data. Bioinformatics.

[CR8] Bu D, Luo H, Huo P, Wang Z, Zhang S, He Z, Wu Y, Zhao L, Liu J, Guo J, Fang S, Cao W, Yi L, Zhao Y, Kong L (2021). KOBAS-i: intelligent prioritization and exploratory visualization of biological functions for gene enrichment analysis. Nucleic Acids Res.

[CR9] Burkett TJ, Garfinkel DJ (1994). Molecular characterization of the *SPT23* gene: a dosage-dependent suppressor of Ty-induced promoter mutations from *Saccharomyces*
*cerevisiae*. Yeast.

[CR10] Camacho C, Coulouris G, Avagyan V, Ma N, Papadopoulos J, Bealer K, Madden TL (2009). BLAST+: architecture and applications. BMC Bioinformatics.

[CR11] Carratu L, Franceschelli S, Pardini CL, Kobayashi GS, Horvath I, Vigh L, Maresca B (1996). Membrane lipid perturbation modifies the set point of the temperature of heat shock response in yeast. Proc Natl Acad Sci U S A.

[CR12] Chappidi S, Villa EC, Cantarel BL (2019). Using Mothur to determine bacterial community composition and structure in 16S ribosomal RNA datasets. Curr Protoc Bioinformatics.

[CR13] Chatterjee MT, Khalawan SA, Curran BPG (1997). Alterations in cellular lipids may be responsible for the transient nature of the yeast heat shock response. Microbiology.

[CR14] Chatterjee MT, Khalawan SA, Curran BPG (2000). Cellular lipid composition influences stress activation of the yeast general stress response element (STRE). Microbiology.

[CR15] De Maeyer D, Renkens J, Cloots L, De Raedt L, Marchal K (2013). PheNetic: network-based interpretation of unstructured gene lists in *E. coli*. Mol Biosyst.

[CR16] De Maeyer D, Weytjens B, Renkens J, De Raedt L, Marchal K (2015). PheNetic: network-based interpretation of molecular profiling data. Nucleic Acids Res.

[CR17] Dennis G, Sherman BT, Hosack DA, Yang J, Gao W, Lane HC, Lempicki RA (2003). DAVID: database for annotation, visualization, and integrated discovery. Genome Biol.

[CR18] DiCarlo JE, Norville JE, Mali P, Rios X, Aach J, Church GM (2013). Genome engineering in *Saccharomyces cerevisiae* using CRISPR-Cas systems. Nucleic Acids Res.

[CR19] Dula ML, Holmes SG (2000). *MGA2* and *SPT23* are modifiers of transcriptional silencing in yeast. Genetics.

[CR20] Esnault C, Lee M, Ham C, Levin HL (2019). Transposable element insertions in fission yeast drive adaptation to environmental stress. Genome Res.

[CR21] Fingerman EG, Dombrowski PG, Francis CA, Sniegowski PD (2003). Distribution and sequence analysis of a novel Ty3-like element in natural *Saccharomyces*
*paradoxus* isolates. Yeast.

[CR22] Fink G, Farabaugh P, Roeder G, Chaleff D (1981). Transposable elements (Ty) in yeast. Cold Spring Harb Symp Quant Biol.

[CR23] Folch J, Lees M, Stanley GHS (1957). A simple method for the isolation and purification of total lipides from animal tissues. J Biol Chem.

[CR24] Garfinkel DJ, Boeke JD, Fink GR (1985). Ty element transposition: reverse transcriptase and virus-like particles. Cell.

[CR25] Grigull J, Mnaimneh S, Pootoolal J, Robinson MD, Hughes TR (2004). Genome-wide analysis of mRNA stability using transcription inhibitors and microarrays reveals posttranscriptional control of ribosome biogenesis factors. Mol Cell Biol.

[CR26] Grishagin IV (2015). Automatic cell counting with ImageJ. Anal Biochem.

[CR27] Hackett EA, Esch RK, Maleri S, Errede B (2006). A family of destabilized cyan fluorescent proteins as transcriptional reporters in *S*. *cerevisiae*. Yeast.

[CR28] Hoppe T, Matuschewski K, Rape M, Schlenker S, Ulrich HD, Jentsch S (2000). Activation of a membrane-bound transcription factor by regulated ubiquitin/proteasome-dependent processing. Cell.

[CR29] Hoshida H, Akada R (2017) High-temperature bioethanol fermentation by conventional and nonconventional yeasts. In: Sibirny A (ed) Biotechnol Yeasts Filamentous Fungi Springer, Cham, 39–61 10.1007/978-3-319-58829-2_2

[CR30] Infante JJ, Dombek KM, Rebordinos L, Cantoral JM, Young ET (2003). Genome-wide amplifications caused by chromosomal rearrangements play a major role in the adaptive evolution of natural yeast. Genetics.

[CR31] Jiang Y, Vasconcelles MJ, Wretzel S, Light A, Martin CE, Goldberg MA (2001). MGA2 is involved in the low-oxygen response element-dependent hypoxic induction of genes in *Saccharomyces*
*cerevisiae*. Mol Cell Biol.

[CR32] Jimoh SO, Ado SA, Ameh JB, Whong CMZ (2013). Heat-shock and ethanol-osmotic effect on fermentable yeast cells. Int J Biol Biol Sci.

[CR33] Kim I, Yun H, Iwahashi H, Jin I (2006). Genome-wide expression analyses of adaptive response against medadione-induced oxidative stress in *Saccharomyces cerevisiae* KNU5377. Process Biochem.

[CR34] Kimura Y, Yashiroda H, Kudo T, Koitabashi S, Murata S, Kakizuka A, Tanaka K (2009). An inhibitor of a deubiquitinating enzyme regulates ubiquitin homeostasis. Cell.

[CR35] Koutelou E, Hirsch CL, Dent SYR (2010). Multiple faces of the SAGA complex. Curr Opin Cell Biol.

[CR36] Li H, Durbin R (2009). Fast and accurate short read alignment with Burrows-Wheeler transform. Bioinformatics.

[CR37] Mager WH, Ferreira PM (1993). Stress response of yeast. Biochem J.

[CR38] Magoč T, Salzberg SL (2011). FLASH: fast length adjustment of short reads to improve genome assemblies. Bioinformatics.

[CR39] Matsushita K, Azuma Y, Kosaka T, Yakushi T, Hoshida H, Akada R, Yamada M (2016). Genomic analyses of thermotolerant microorganisms used for high-temperature fermentations. Biosci Biotechnol Biochem.

[CR40] McKenna A, Hanna M, Banks E, Sivachenko A, Cibulskis K, Kernytsky A, Garimella K, Altshuler D, Gabriel S, Daly M, DePristo MA (2010). The genome analysis toolkit: a mapreduce framework for analyzing next-generation DNA sequencing data. Genome Res.

[CR41] Moraitis C, Curran BPG (2004). Reactive oxygen species may influence the heat shock response and stress tolerance in the yeast *Saccharomyces*
*cerevisiae*. Yeast.

[CR42] Moraitis C, Curran BPG (2007). Can the different heat shock response thresholds found in fermenting and respiring yeast cells be attributed to their differential redox states?. Yeast.

[CR43] Mourier T, Willerslev E (2010). Large-scale transcriptome data reveals transcriptional activity of fission yeast LTR retrotransposons. BMC Genomics.

[CR44] Panaretou B, Piper P (2006) Isolation of yeast plasma membranes. In: Xiao W (ed) Yeast protocols. vol 313, Methods in Molecular Biology. Humana Press, Totowa, NJ, 27–32 10.1385/1-59259-958-3:027 10.1385/1-59259-958-3:027 16118421

[CR45] Paquin CE, Williamson VM (1984). Temperature effects on the rate of Ty transposition. Science.

[CR46] Piper PW (1995). The heat shock and ethanol stress responses of yeast exhibit extensive similarity and functional overlap. FEMS Microbiol Lett.

[CR47] Rape M, Hoppe T, Gorr I, Kalocay M, Richly H, Jentsch S (2001). Mobilization of processed, membrane-tethered SPT23 transcription factor by CDC48UFD1/NPL4, a ubiquitin-selective chaperone. Cell.

[CR48] Raskin A, Lange S, Banares K, Lyon RC, Zieseniss A, Lee LK, Yamazaki KG, Granzier HL, Gregorio CC, McCulloch AD, Omens JH, Sheikh F (2012). A novel mechanism involving four-and-a-half LIM domain protein-1 and extracellular signal-regulated kinase-2 regulates titin phosphorylation and mechanics. J Biol Chem.

[CR49] Robinson MD, McCarthy DJ, Smyth GK (2010). edgeR: a Bioconductor package for differential expression analysis of digital gene expression data. Bioinformatics.

[CR50] Ruiz-Roig C, Noriega N, Duch A, Posas F, de Nadal E (2012). The Hog1 SAPK controls the Rtg1/Rtg3 transcriptional complex activity by multiple regulatory mechanisms. Mol Biol Cell.

[CR51] Sakaki K, Tashiro K, Kuhara S, Mihara K (2003). Response of genes associated with mitochondrial function to mild heat stress in yeast *Saccharomyces*
*cerevisiae*. J Biochem.

[CR52] Sanz AB, García R, Rodríguez-Peña JM, Nombela C, Arroyo J (2016). Cooperation between SAGA and SWI/SNF complexes is required for efficient transcriptional responses regulated by the yeast MAPK Slt2. Nucleic Acids Res.

[CR53] Schloss PD (2020). Reintroducing mothur: 10 years later. Appl Environ Microbiol.

[CR54] Servant G, Pennetier C, Lesage P (2008). Remodeling yeast gene transcription by activating the Ty1 long terminal repeat retrotransposon under severe adenine deficiency. Mol Cell Biol.

[CR55] Singer T, Burke E (2003) High-throughput TAIL-PCR as a tool to identify DNA flanking insertions. In: Grotewold E (ed) Plant functional genomics. vol 236, Methods in Molecular Biology™. Humana Press, 241–271 10.1385/1-59259-413-1:241 10.1385/1-59259-413-1:241 14501069

[CR56] Swan TM, Watson K (1998). Stress tolerance in a yeast sterol auxotroph: role of ergosterol, heat shock proteins and trehalose. FEMS Microbiol Lett.

[CR57] Thompson JD, Gibson TJ, Higgins DG (2002) Multiple sequence alignment using ClustalW and ClustalX. Curr Protoc Bioinformatics Chapter 2:Unit 2.3. 10.1002/0471250953.bi0203s00 10.1002/0471250953.bi0203s00 18792934

[CR58] Wade CH, Umbarger MA, McAlear MA (2006). The budding yeast rRNA and ribosome biosynthesis (RRB) regulon contains over 200 genes. Yeast.

[CR59] Wang Y, Gibney PA, West JD, Morano KA (2012). The yeast Hsp70 Ssa1 is a sensor for activation of the heat shock response by thiol-reactive compounds. Mol Biol Cell.

[CR60] Watson K, Cavicchioli R (1983). Acquisition of ethanol tolerance in yeast cells by heat shock. Biotechnol Lett.

[CR61] Wingender E, Chen X, Fricke E, Geffers R, Hehl R, Liebich I, Krull M, Matys V, Michael H, Ohnhäuser R, Prüss M, Schacherer F, Thiele S, Urbach S (2001). The TRANSFAC system on gene expression regulation. Nucleic Acids Res.

[CR62] Winzeler EA, Castillo-Davis CI, Oshiro G, Liang D, Richards DR, Zhou Y, Hartl DL (2003). Genetic diversity in yeast assessed with whole-genome oligonucleotide arrays. Genetics.

[CR63] Wolf DA, Petroski MD (2009). Rfu1: stimulus for the ubiquitin economy. Cell.

[CR64] Xie X, Ma X, Liu Y-G (2019) Decoding Sanger sequencing chromatograms from CRISPR-induced mutations. In: Qi Y (ed) Plant genome editing with CRISPR Systems. vol 1917, Methods Mol Biol Humana Press, New York, NY, 33–43 10.1007/978-1-4939-8991-1_3 10.1007/978-1-4939-8991-1_3 30610626

[CR65] Yoshioka K, Hashimoto N (1983). Cellular fatty acid and ester formation by brewers′ yeast. Agric Biol Chem.

[CR66] Zhang S, Burkett TJ, Yamashita I, Garfinkel DJ (1997). Genetic redundancy between *SPT23* and *MGA2*: regulators of Ty-induced mutations and Ty1 transcription in *Saccharomyces*
*cerevisiae*. Mol Cell Biol.

[CR67] Zhang S, Skalsky Y, Garfinkel DJ (1999). *MGA2* or *SPT23* is required for transcription of the Δ9 fatty acid desaturase gene, *OLE1*, and nuclear membrane integrity in *Saccharomyces*
*cerevisiae*. Genetics.

