# Supplementary material for: Role of spt23 in Saccharomyces cerevisiae thermal tolerance
Source: Appl Microbiol Biotechnol. 2022 Apr 27;106(9-10):3691–705. doi: 10.1007/s00253-022-11920-3 (PMC9151549; doi:10.1007/s00253-022-11920-3)
Supplement: Supplementary file 1 — Supplementary file1 (PDF 2630 KB) [file 253_2022_11920_MOESM1_ESM.pdf]

Applied Microbiology and Biotechnology

Role of *spt23* in *Saccharomyces cerevisiae* thermal tolerance

Zhilong Lu[1,2,3] • Yanling Wu[3] • Ying Chen[3] • Xiaoling Chen[3] • Renzhi Wu[3] • Qi Lu[3] • Dong Chen[3] • Ribo Huang[1,2,3]

✉ Ribo Huang

rbhuang_gxu@163.com

Fax +867702503990

1 State Key Laboratory for Conservation and Utilization of Subtropical Agro-Bioresources, Guangxi University, Nanning, Guangxi 530004, P. R. China 530004, P. R. China

2 College of Life Science and Technology, Guangxi University, Nanning, Guangxi 530004, P. R. China

3 National Engineering Research Center for Non-Food Biorefinery, Guangxi Academy of Sciences, Nanning, Guangxi 530007, P. R. China

Table S1 PCR primers used for real-time fluorescence quantitative PCR of key genes involved in the functional response to stress

Gene	Primer	Sequence (5'-3')	Product length (bp)
alg9	ALG9-F	CCAGGCAATGTCACGGATAG	163
	ALG9-R	TGGCAGCAGGAAAGAACTTG	
dog2	DOG2-F	GCTATCGTTACCTCTGGTTCTC	120
	DOG2-R	ATCAGGCTTACCGTTCTTCAC	
stf2	STF2-F	GTGGACCGAACGTGAAGGAA	130
	STF2-R	AATCTCATCGCCTGGCTTACC	
rfu1	RFU1-F	TCGTCGTCATCGTCGTCATC	187
	RFU1-R	CGGGAGTTGTGTTTGCGTTTA	
spt23	SPT23-F	ACCGCAATGATGAACAACAACA	138
	SPT23-R	GGACGACCAAGAGTCTAGTGAA	
erg1	ERG1-F	ATGAAGCCGTCAGCCAAGG	102
	ERG1-R	TTAGAGCGTCACCGATAACACA	
ole1	OLE1-F	AATTCACATCTCCGAACAACCA	177
	OLE1-R	CCACCGACAGCGTAGTAGAA	
mga2	MGA2-F	TTGGATGTAGAGGTCTCAGGTT	126
	MGA2-R	AATGGAATCGCTAGGCAAGTAA	
swi5	SWI5-F	GCAACAACAACTACAGGCTACC	165
	SWI5-R	GGTGACCCAGTGAACGAAGTAT	
acc1	ACC1-F	GCCAAGCGTAATATGCCAGTT	121
	ACC1-R	TCTCACGACCTTCAATCATCCA	

Table S2 Primers used to amplify LTRs and TYs

Primer	Sequence (5'->3')
ltr1-f	TCGTCGGCAGCGTCAGATGTGTATAAGAGACA
	GGAATAAAAATCCACTATCGTCTATC
ltr1-r	GTCTCGTGGGCTCGGAGATGTGTATAAGAGACA
	GTGTTGATAAAGGCTATAATATTAGG
ltr2-f	TCGTCGGCAGCGTCAGATGTGTATAAGAGACA
	GTTAATAAGGCAATAATATTAGGTATGTAG
ltr2-r	GTCTCGTGGGCTCGGAGATGTGTATAAGAGACA
	GGTTGGAATAAAAATCAACTATCATC
ltr3-f	TCGTCGGCAGCGTCAGATGTGTATAAGAGACAGTGTAT
	CTCAAAATGAGATATGTC
ltr3-r	GTCTCGTGGGCTCGGAGATGTGTATAAGAGACAGT
	GTATTACGGGCTCGAGTAATAC
ltr4-r	GTCTCGTGGGCTCGGAGATGTGTATAAGAGACA
	GTGATAATTAGAGGTAAAAAATTAG
ltr4_f	TCGTCGGCAGCGTCAGATGTGTATAAGAGACA
	GAACGAGAGTAATTGATAGTGACATG
ltr5-f	TCGTCGGCAGCGTCAGATGTGTATAAGAGACAGAT
	AGCATGATATGAGTAATGCTTTAG
ltr5-r	GTCTCGTGGGCTCGGAGATGTGTATAAGAGACAGT
	GACGTAGTGAATTACGATCTAAC
ty1_sf	TCGTCGGCAGCGTCAGATGTGTATAAGAGACA
	GAGTATCCATCATCAGTTGGAACG
ty1-sr	GTCTCGTGGGCTCGGAGATGTGTATAAGAGACA
	GCGTACGGGTTTTCCGTTTACTGTC
ty2_sf	TCGTCGGCAGCGTCAGATGTGTATAAGAGACA
	GGTCTTTTGATTTCACCCTGGTC
ty2_sr	GTCTCGTGGGCTCGGAGATGTGTATAAGAGACA

	GATCCAATCGATTTAAAAAATCAACAC
ty3-sf	TCGTCGGCAGCGTCAGATGTGTATAAGAGACAG
	CACAAACCAGAGACATTAAAAGACG
ty3_sr	GTCTCGTGGGCTCGGAGATGTGTATAAGAGACA
	GTTCTTACAATAGAAGCATAGCCG
ty4_sf	TCGTCGGCAGCGTCAGATGTGTATAAGAGACA
	GGACATTTTTAGGATAATCGATGG
ty4_sr	GTCTCGTGGGCTCGGAGATGTGTATAAGAGACA
	GTCTTAAGGATTCATGTAAGGTTCC
ty5_sf	TCGTCGGCAGCGTCAGATGTGTATAAGAGAC
	AGCTAAACTCAACCATGGATGAGC
ty5_sr	GTCTCGTGGGCTCGGAGATGTGTATAAGAGACA
	GATTGAGTATAATTTCGTTAATTCTTGC

Table S3 SNP and Indel distribution along chromosomes of strain 1015

Chromo-some	SNP + Indel	SNP	Non-synonymous SNP	Synonymous SNP	Indel	Insertion	Deletion
I	1468	1410	629	781	58	18	40
II	3815	3636	777	2859	179	48	131
III	1963	1859	715	1144	104	34	70
IV	9877	9472	4328	5144	405	131	274
V	3696	3519	1313	2206	177	61	116
VI	1897	1813	1067	746	84	34	50
VII	6809	6505	2506	3999	304	107	197
VIII	3261	3123	1382	1741	138	39	99
IX	2903	2783	1589	1194	120	41	79
X	4114	3945	2007	1938	169	49	120
XI	4141	3974	1932	2042	167	53	114
XII	5583	5358	2874	2484	225	65	160
XIII	4547	4330	852	3478	217	51	166
XIV	5049	4836	1887	2949	213	66	147
XV	6948	6667	3519	3148	281	99	182
XVI	6198	5964	3123	2841	234	75	159
Mito	217	127	0	127	90	33	57
Total	72486	69321	30500	38821	3165	1004	2161

Table S4 SNP and Indel distribution along chromosomes of strain 101530

Chromo-some	SNP + Indel	SNP	Non-synonymous SNP	Synonymous SNP	Indel	Insertion	Deletion
I	985	958	415	543	27	1	26
II	3380	3223	589	2634	157	63	94
III	1378	1295	447	848	83	41	42
IV	7743	7398	2582	4816	345	179	166
V	3010	2853	950	1903	157	74	83
VI	1188	1116	582	534	72	39	33
VII	5685	5420	1994	3426	265	126	139
VIII	2795	2681	1191	1490	114	51	63
IX	2262	2152	1209	943	110	60	50
X	3503	3348	1649	1699	155	72	83
XI	3522	3367	1569	1798	155	82	73
XII	4686	4472	2254	2218	214	104	110
XIII	4116	3902	739	3163	214	101	113
XIV	3964	3786	1450	2336	178	79	99
XV	5977	5724	2900	2824	253	121	132
XVI	5220	4993	2435	2558	227	110	117
Mito	79	28	0	28	51	20	31
Total	59519	56716	22955	33761	2803	1349	1454

Table S5 KEGG and Reactome pathway enrichment analysis of differentially expressed genes when comparing strain 101530 grown at 30°C and 37°C for 16 h

**KO term**	**ID**	**N***	**B***	***p****
Ribosome	sce03010	86	183	0.004
Metabolic pathways	sce01100	259	754	0.023
**Reactome term**	**ID**	**N***	**B***	***p****
Formation of a pool of free 40S subunits	R-SCE-72689	60	108	0.004
GTP hydrolysis and joining of the 60S ribosomal subunit	R-SCE-72706	59	108	0.004
SRP-dependent cotranslational protein targeting to membrane	R-SCE-1799339	60	113	0.004
Metabolism of RNA	R-SCE-8953854	108	248	0.004
Major pathway of rRNA processing in the nucleolus and cytosol	R-SCE-6791226	40	63	0.004
rRNA processing in the nucleus and cytosol	R-SCE-8868773	40	63	0.004
rRNA processing	R-SCE-72312	40	63	0.004
NMD independent of the EJC	R-SCE-975956	60	115	0.004
NMD	R-SCE-927802	62	123	0.005
NMD enhanced by the EJC	R-SCE-975957	62	123	0.005
Eukaryotic translation initiation	R-SCE-72613	62	130	0.015
Cap-dependent translation initiation	R-SCE-72737	61	128	0.016
Translation	R-SCE-72766	65	148	0.046

N*: input number; B*: background number; *p**: corrected *p*-value

Table S6 KEGG and Reactome pathway enrichment analysis of differentially expressed genes when comparing strain 1015 grown at 30°C and 37°C for 16 h

**KO term**	**ID**	**N***	**B***	***p****
Ribosome	sce03010	68	183	0.001
**Reactome term**	**ID**	**N***	**B***	***p****
Formation of a pool of free 40S subunits	R-SCE-72689	43	108	0.013
GTP hydrolysis and joining of the 60S ribosomal subunit	R-SCE-72706	42	108	0.015
SRP-dependent co-translational protein targeting to membrane	R-SCE-1799339	43	113	0.015
Nonsense mediated decay (NMD) independent of the exon junction complex (EJC)	R-SCE-975956	42	115	0.025
L13a-mediated translational silencing of ceruloplasmin expression	R-SCE-156827	44	123	0.025
Cap-dependent translation initiation	R-SCE-72737	45	128	0.025
Eukaryotic translation initiation	R-SCE-72613	45	130	0.029
Translation	R-SCE-72766	49	148	0.033
NMD	R-SCE-927802	42	123	0.041
NMD enhanced by the EJC	R-SCE-975957	42	123	0.041

N*: input number; B*: background number; *p**: corrected *p*-value

Table S7 KEGG and Reactome pathway enrichment analysis of differentially expressed genes when comparing strain 1015 grown at 30°C and 37°C for 40 h

**KO term**	**ID**	**N***	**B***	***p****
-	-	-	-	-
**Reactome term**				
Major pathway of rRNA processing in the nucleolus and cytosol	R-SCE-6791226	39	63	5.70E-05
rRNA processing in the nucleus and cytosol	R-SCE-8868773	39	63	5.70E-05
rRNA processing	R-SCE-72312	39	63	5.70E-05

*N: input number; B: background number; *p*: corrected *p*-value

Table S8 H-CORE, core gene set for heat-shock response independent of strain and growth phase

H-CORE（258）
YHR089C,YBL090W,snR50,YER009W,YOL037C,YIL048W,YOL033W,<br>YOL014W,YNL134C,YLR196W,YOR120W,YLR224W,YIL093C,YMR033W,<br>YIL144W,YGR008C,YDR268W,YDL184C,YLR090W,YLL034C,YPL153C,<br>YBR246W,YMR122W-YPL211W,YAR033W,YCR071C,YDR317W,<br>YDR280W,YHR088W,YNL056W,YIL067C,YER185W,YDR375C,YHL048W,<br>YDR381C-A,YJL180C,YMR058W, YDR511W,YIL095W,YCR096C,YKR069W<br>YKL167C,YER078C,YDL191W,YKR024C,YJR080C,YBR146W,YDR464W,<br>YGL071W,YOL038C-A,YGL040C,YGL143C,YLL011W,YIL056W,YGR218W,<br>YOR029W,YLR455W,YDR031W,YOR044W,YOL147C,YCR003W,YHR036W,<br>YEL057C,YGL259W,YML024W,YGR084C,YKR045C,YGR059W,YAL037C-,<br>YDR493W,YFR040W,YIL070C,YNL179C,YNL079C,YEL007W,YOR047C,<br>YLR110C,YCL048W,YJR156C,YLR056W,YOR077W,YJR096W,YML028W,<br>YJR064W,YDR466W,YIL146C,YHL024W,YDR116C,YPL104W,YLR108C,<br>YBR096W,YJL069C,YDL230W,YOR014W,YDR376W,YDR111C,YDR033W,<br>YDR034C-A,YLR180W,YOL082W,YOR045W,YER053C-A,YJL003W,<br>YIL013C,YGL035C,YIL055C,YMR251W,YBR185C,YER119C-A,YBR296C,<br>YJL063C,YDR412W,YOR027W,YNL081C,YDR310C,YHR038W,YML100W-A,<br>YML052W,YGR215W,YDR050C,YGR054W,YEL072W,YLR278C,YJL062W-A,<br>YGL205W,YLR129W,YGR019W,YPR110C,YMR144W,YNL133C,YLR134W,<br>YFR001W,YIL057C,YHR068W,YHR018C,YMR015C,YCR057C,YDL003W,

YLR448W,YNL281W,YHR168W,YJR118C,YER062C,YNR022C,YDR359C,
YMR185W,YJR135W-,YMR096W,YNR008W,YDR276C,YER042W,YGL101W,
YLR011W,YGL014W,YFL024C,snR67,YNL300W,YML058W-A,YPL149W,
YBR275C,YBR297W,YGR132C,YMR199W,YMR267W,YMR032W,YAL005C,
YIL098C,YGR292W,YIL066C,YGR249W,YEL069C,YER181C,YBR296C,
YMR113W,YPL128C,YJL060W,YGR174C,YGR208W,YLR289W,YDL196W,
YLR287C-A,YKL084W,YER084W,YPR167C,YBR230W-A,YCR072C,YBR200W-A
YBL098W,YKL171W,YHR169W,YNL010W,YPL098C,YKL194C,YDR430C,
YMR244W,YJL109C,YNL070W,YOR150W,YJL096W,YBR222C,YML090W,
YIL164C,YLL051C,YJR122W,YGL171W,YOL055C,snR37,YFL056C,YHR148W,
YDR502C,YDL247W,YDR089W,YHR024C,YNL142W,YGL215W,YNR014W,
YDR259C,YOR176W,YIL153W,YDR321W,YMR060C,YDL246C,YPL130W,
YLR177W,YPR166C,YHL033C,YLL009C,YER026C,YJL050W,YNL075W,
YKR052C,YJR104C,YJR040W,YLR084C,YMR271C,YKR010C,YPR145C-A,
YHR159W,YJR160C,YLR222C,YDL210W,YOR382W,YCR098C,YNL090W,
YGL096W,YOR158W,YJR003C,YIL157C,YGL070C,YLL028W,YDL119C,
YNL251C,YBL100C,YLL053C,YKR066C,YHR005C-A,YNL175C,YGR234W

Table S9. Ten most influential drive mutants at 16 and 40 h based on detection with the PheNetic program.

16 h		40 h	
Gene	Rank	Gene	Rank
spt23	1	*swi4*	1
fhl1	1	*spt23*	2
cbf1	2	*cbf1*	2
swi4	3	*sgv1*	3
sko1	3	*fkh2*	4
gre1	4	*fhl1*	4
dal81	5	*yor338w*	5
flo8	6	*rtg3*	6
gcn4	6	*dal81*	7
fkh2	7	*sko1*	8

Table S10 M-CORE, *spt23*-related key mutant gene set

S-CORE,439
YAL004W, YAL005C, YAL045C, YAR068W, YBL007C, YBL029C-A, YBL029W, YBL033C, YBL048W, YBL053W, YBL062W, YBL089W, YBL103C, YBR033W, YBR051W, YBR056W, YBR074W, YBR078W, YBR085C-A, YBR092C, YBR099C, YBR113W, YBR134W, YBR138C, YBR147W, YBR196C-A, YBR204C, YBR209W, YBR230W-A, YBR255W, YBR259W, YCL041C, YCL042W, YCR006C, YCR013C, YCR019W, YCR025C, YDL009C, YDL023C, YDL026W, YDL027C, YDL037C, YDL039C, YDL085C-A, YDL094C, YDL129W, YDL140C, YDL150W, YDL151C, YDL156W, YDL164C, YDL169C, YDL170W, YDL183C, YDL213C, YDL221W, YDL222C, YDL243C, YDL244W, YDR008C, YDR010C, YDR011W, YDR034W-B, YDR084C, YDR089W, YDR112W, YDR114C, YDR125C, YDR133C, YDR179W-A, YDR187C, YDR209C, YDR222W, YDR230W, YDR241W, YDR246W-A, YDR271C, YDR274C, YDR281C, YDR282C, YDR310C, YDR317W, YDR350C, YDR370C, YDR381C-A, YDR417C, YDR426C, YDR442W, YDR476C, YDR491C, YDR493W, YDR509W, YDR512C, YDR522C, YDR524C-B, YDR526C, YDR535C, YDR540C, YDR541C, YEL008W, YEL009C, YEL035C, YEL040W, YEL059W, YEL069C, YEL072W, YEL073C, YER034W, YER046W, YER053C-A, YER058W, YER060W, YER076C, YER080W, YER085C, YER109C, YER111C, YER137C, YER175C, YER180C, YER184C, YER185W, YFL010W-A, YFL019C, YFL055W, YFR024C-A, YFR035C, YFR045W, YGL007W, YGL072C, YGL088W, YGL101W, YGL102C, YGL107C, YGL117W, YGL118C, YGL119W, YGL136C, YGL149W, YGL165C, YGL186C, YGL188C, YGL217C, YGL218W, YGL255W, YGL257C, YGL263W, YGR001C, YGR008C, YGR015C, YGR031C-A, YGR042W, YGR044C, YGR050C, YGR051C, YGR053C, YGR063C, YGR109W-B, YGR110W, YGR122W, YGR131W, YGR137W, YGR154C, YGR160W, YGR190C, YGR212W, YGR230W, YGR250C, YGR288W, YHL016C, YHL026C, YHL028W, YHL038C, YHL040C, YHL049C, YHL050C, YHR007C-A, YHR017W, YHR043C, YHR049W, YHR079C, YHR139C, YHR143W, YHR150W, YHR151C, YHR159W, YHR165C, YHR201C, YIL011W, YIL014W, YIL046W-A, YIL049W, YIL060W, YIL073C, YIL082W-A, YIL096C, YIL119C, YIL131C, YIL164C, YIR023W, YIR028W, YJL005W, YJL009W, YJL015C, YJL032W, YJL037W, YJL038C, YJL051W, YJL077C, YJL077W-B, YJL105W, YJL116C, YJL133C-A, YJL145W, YJL171C, YJL175W, YJL188C, YJL191W, YJL203W, YJL206C, YJL211C, YJL212C, YJR003C, YJR008W, YJR018W, YJR020W, YJR027W, YJR054W, YJR060W, YJR079W, YJR087W, YJR100C, YJR107W, YJR111C, YJR114W, YJR120W, YJR121W, YJR124C, YJR127C, YJR129C, YJR151C, YJR154W, YKL020C, YKL030W, YKL031W, YKL036C, YKL046C, YKL063C, YKL071W, YKL083W, YKL094W, YKL096C-B, YKL096W, YKL096W-A, YKL133C, YKL147C, YKL162C, YKL170W, YKL172W, YKL187C, YKL195W, YKL219W, YKR004C, YKR015C, YKR040C, YKR041W, YKR056W, YKR073C, YKR102W, YKR104W, YLL018C-A, YLR020C, YLR037C, YLR041W, YLR047C, YLR049C, YLR062C, YLR073C, YLR076C, YLR094C, YLR107W, YLR126C, YLR130C, YLR152C, YLR171W, YLR214W, YLR251W, YLR279W, YLR296W, YLR316C, YLR331C, YLR338W, YLR346C, YLR361C, YLR375W, YLR385C, YLR399C, YLR429W, YLR460C, YLR466W, YML003W, YML050W, YML053C, YML101C-A, YML118W, YML119W, YML128C, YMR007W, YMR045C, YMR063W, YMR085W, YMR103C, YMR107W, YMR122C, YMR122W-A, YMR130W, YMR135W-A, YMR141C, YMR172C-A, YMR173W-A, YMR182W-A,

YMR193C-A, YMR244C-A, YMR245W, YMR251W, YMR251W-A, YMR265C, YMR285C, YMR290W-A, YMR304C-A, YMR306C-A, YMR320W, YNL011C, YNL018C, YNL025C, YNL043C, YNL046W, YNL057W, YNL063W, YNL068C, YNL080C, YNL105W, YNL108C, YNL113W, YNL115C, YNL142W, YNL144C, YNL162W-A, YNL167C, YNL172W, YNL174W, YNL190W, YNL193W, YNL195C, YNL213C, YNL266W, YNL270C, YNL296W, YNL322C, YNL331C, YNL335W, YNR014W, YNR061C, YNR067C, YNR070W, YOL019W, YOL035C, YOL047C, YOL048C, YOL050C, YOL052C-A, YOL064C, YOL084W, YOL085C, YOL089C, YOL099C, YOL101C, YOL119C, YOL124C, YOL125W, YOL150C, YOL152W, YOL158C, YOR011W, YOR019W, YOR030W, YOR031W, YOR032C, YOR076C, YOR082C, YOR084W, YOR087W, YOR114W, YOR128C, YOR134W, YOR139C, YOR152C, YOR162C, YOR169C, YOR222W, YOR237W, YOR274W, YOR277C, YOR291W, YOR313C, YOR338W, YOR343C, YOR344C, YOR365C, YOR377W, YOR378W, YOR382W, YOR383C, YOR391C, YPL067C, YPL123C, YPL159C, YPL197C, YPL205C, YPL221W, YPL223C, YPL224C, YPL272C, YPR005C, YPR009W, YPR012W, YPR013C, YPR025C, YPR036W-A, YPR038W, YPR044C, YPR092W, YPR104C, YPR126C, YPR150W, YPR157W, YPR161C, YPR196W, YPR202W

Table S11 Cell membrane lipid acid content (%) determination for strains 1015 and 101530 under different growth temperature conditions

LAs	REF30[a]	REF37[a]	WT30[b]	WT37[b]	Del30[c]	Del37[c]	IE30[d]	IE37[d]
C15:1n5	23.4	0	0	10.7	0	0	0	0
C16:0	43.5	61.8	38	30.5	40.9	21.1	46.6	39.4
C16:1	0	0	14.1	0	23.4	40.1	0	0
C18:0	0	0	0	0	0	6.7	0	0
C18:1	0	0	16.3	25.5	0	23.7	0	0
C18:2	0	0	0	10.7	0	0	0	0
C20:4	33.1	38.2	31.6	22.4	35.7	8.5	53.4	60.6
SLA	43.5	61.8	54.3	56.1	40.9	44.7	46.6	39.4
ULA	56.5	38.2	45.7	43.9	59.1	55.3	53.4	60.6

[a]Reference, WT (wild-type) of strain1015

[b]WT (wild-type) of strain101530

[c]Del (*spt23* deleted) type of strain 101530

[d]IE (*spt23* integrative expressed) type of strain 101530

Table S12 LTR diversity metrics for strains 1015 and 101530 under different growth temperature conditions

Strain	Shannon	Chao	Shannon evenness	OTUs
WT30[a]	5.48	480.67	0.90	69
WT37[a]	5.30	450.75	0.90	55
REF30[b]	5.59	1180.77	0.87	64
REF37[b]	5.27	552.89	0.90	35
Del30[c]	5.42	497.79	0.89	61
Del37[c]	5.37	372.50	0.92	44
IE30[d]	5.16	366.50	0.90	50
IE37[d]	5.62	545.47	0.91	53

[a]Reference, WT (wild-type) of strain1015

[b]WT (wild-type) of strain101530

[c]Del (*spt23* deleted) type of strain 101530

[d]IE (*spt23* integrative expressed) type of strain 101530

Table S13 T-CORE: *spt23*-TY regulated core gene set

（T-CORE），179
YAR010C，YBL005W，YBL005W-A，YBL005W-B，YBR012W-A，YBR012W-B, YLL039C, YKR081C,
YPL240C, YOL030W, YNL258C, YKL049C, YKL007W, YNR008W, YHL049C, YGR035C, YOL083W, YOL082W,
YGL122C, YGL209W, YPR149W, YOL157C, YOL156W, YML125C, YFR028C, YOR344C, YLR022C, YJL082W,
YER151C, YDR510W, YJR010C-A, YGR281W, YDR469W, YLR116W, YDR415C, YIL148W, YDR388W, YKL008C,
YAR010C，YBL005W，YBL005W-A，YBL005W-B，YBR012W-A，YBR012W-BYLL039C, YKR081C,
YPL240C, YOL030W, YNL258C, YKL049C, YKL007W, YNR008W, YHL049C, YGR035C, YOL083W, YOL082W,
YGL122C, YGL209W, YPR149W, YOL157C, YOL156W, YML125C, YFR028C, YOR344C, YLR022C, YJL082W,
YER151C, YDR510W, YJR010C-A, YGR281W, YDR469W, YLR116W, YDR415C, YIL148W, YDR388W, YKL008C,
YPL135W, YPL068C, YPL062W, YOR349W, YOR348C, YOR153W, YNL231C, YNL160W, YMR251W-A,
YMR195W, YMR194C-A, YMR136W, YLR438W, YLL028W, YIL056W, YGR280C, YGR161C, YGR050C,
YER189W, YDR073W, YDR072C, YDR055W, YDR054C, YDR011W, YDR010C, YCR081W, YBR296C, YBL029W,
YAL061W, YAL005C, YAL003W, YLR437C, YJL219W, YIL119C, YBL042C, YBL041W, YNL031C, YOR142W-A,
YMR258C, YDR369C, YDR176W, YDR345C, YOL031C, YNR051C, YOR327C, YLR100W, YLR113W, YKL013C,
YDR468C, YPL283C, YNL337W, YLL066C, YLL065W, YGR296W, YFL064C, YFL063W, YOR151C, YDR007W,
YKL012W, YPL061W, YDR416W, YDL095W, YOR204W, YMR291W, YMR246W, YHR064C, YDR297W, YPR161C,
YPR164W, YOR148C, YCL041C, YCL040W, YML131W, YFR027W, YGL035C, YDR405W, YMR193W, YBL011W,
YKL077W, YGL194C, YCR010C, YCR009C, YIL099W, YOR183W, YOR182C, YMR272C, YJR007W, YIL172C,
YIL171W, YGR249W, YDR133C, YBR149W, YBL043W, YCR088W, YOR018W, YNR019W, YCL019W, YJR135C,
YIL043C, YGL167C, YDR044W, YBR264C, YOL051W, YBR200W, YLR099C, YOL010W, YDR404C, YPL241C,
YDR074W, YMR102C, YDL248W, YDR025W, YOR328W, YOR233W, YOR152C, YDR406W, YDR006C, YDL020C,
YOR178C, YBL103C, YML093W, YDR009W, YBR012W-B, YBR010W, YMR103C, YML097C, YBL102W,
YNR007C, YBL032W, YDR093W, YLR346C, YBR012W-A, YBR008C, YBL005W-B, YBL005W-A, YHR214W,
YDR008C, YBL006C, YBL005W, YBL004W, YAR010C, YBL007C, YOL029C, YGR039W, YDR361C, YML096W,
YDR401WYPL135W, YPL068C, YPL062W, YOR349W, YOR348C, YOR153W, YNL231C, YNL160W, YMR251W-A,
YMR195W, YMR194C-A, YMR136W, YLR438W, YLL028W, YIL056W, YGR280C, YGR161C, YGR050C,
YER189W, YDR073W, YDR072C, YDR055W, YDR054C, YDR011W, YDR010C, YCR081W, YBR296C, YBL029W,

YAL061W, YAL005C, YAL003W, YLR437C, YJL219W, YIL119C, YBL042C, YBL041W, YNL031C, YOR142W-A, YMR258C, YDR369C, YDR176W, YDR345C, YOL031C, YNR051C, YOR327C, YLR100W, YLR113W, YKL013C, YDR468C, YPL283C, YNL337W, YLL066C, YLL065W, YGR296W, YFL064C, YFL063W, YOR151C, YDR007W, YKL012W, YPL061W, YDR416W, YDL095W, YOR204W, YMR291W, YMR246W, YHR064C, YDR297W, YPR161C, YPR164W, YOR148C, YCL041C, YCL040W, YML131W, YFR027W, YGL035C, YDR405W, YMR193W, YBL011W, YKL077W, YGL194C, YCR010C, YCR009C, YIL099W, YOR183W, YOR182C, YMR272C, YJR007W, YIL172C, YIL171W, YGR249W, YDR133C, YBR149W, YBL043W, YCR088W, YOR018W, YNR019W, YCL019W, YJR135C, YIL043C, YGL167C, YDR044W, YBR264C, YOL051W, YBR200W, YLR099C, YOL010W, YDR404C, YPL241C, YDR074W, YMR102C, YDL248W, YDR025W, YOR328W, YOR233W, YOR152C, YDR406W, YDR006C, YDL020C, YOR178C, YBL103C, YML093W, YDR009W, YBR012W-B, YBR010W, YMR103C, YML097C, YBL102W, YNR007C, YBL032W, YDR093W, YLR346C, YBR012W-A, YBR008C, YBL005W-B, YBL005W-A, YHR214W, YDR008C, YBL006C, YBL005W, YBL004W, YAR010C, YBL007C, YOL029C, YGR039W, YDR361C, YML096W, YDR401W

Supplementary Figures

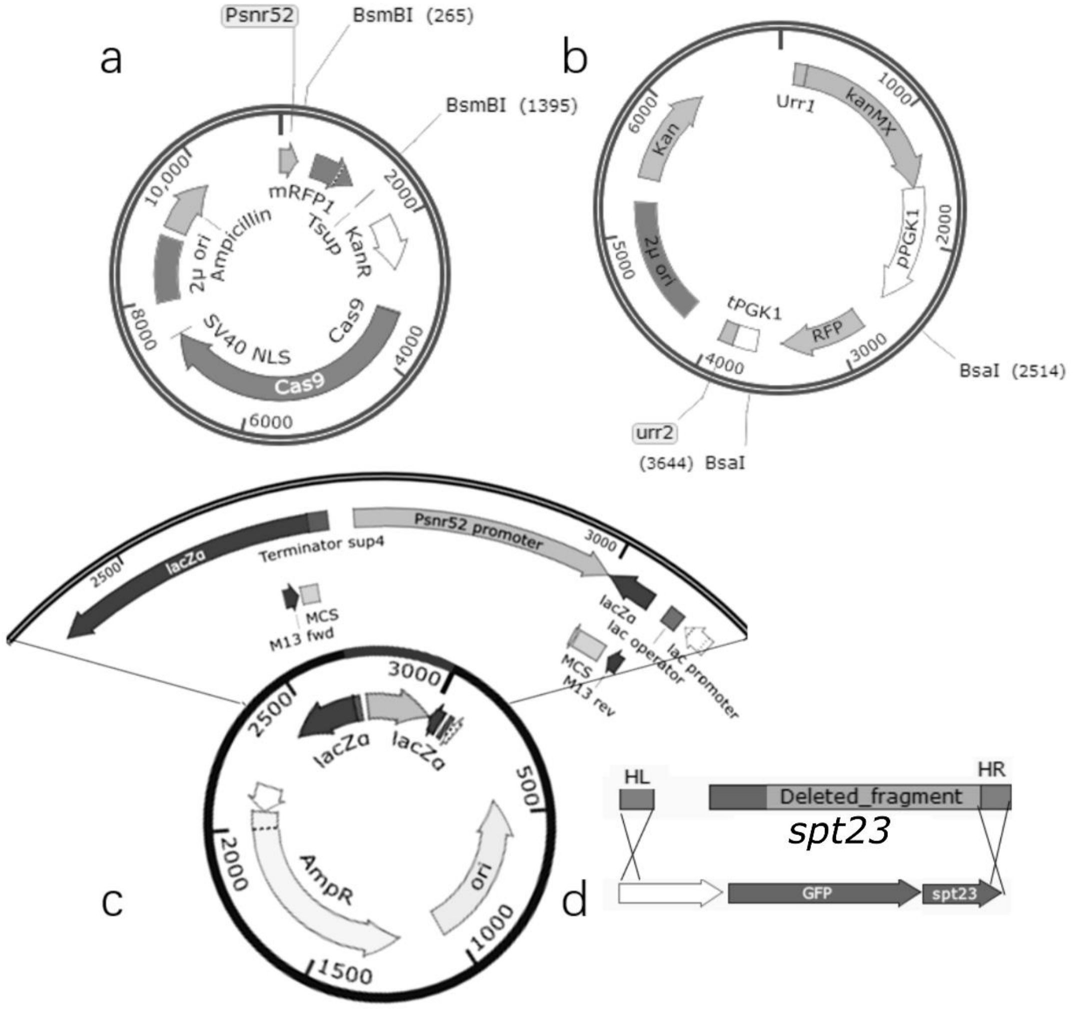

Fig. S1 Plasmids used for *spt23* manipulation. **a**. Schematic of the gene-editing plasmid pCrispr-con that is used for industrial *Saccharomyces cerevisiae* engineering. **b**. pPH4, an expression vector for *Saccharomyces cerevisiae*. **c**. Helper plasmid pMD-gp1. **d**. schematic of integration of *gfp*-*spt23* into the genome.

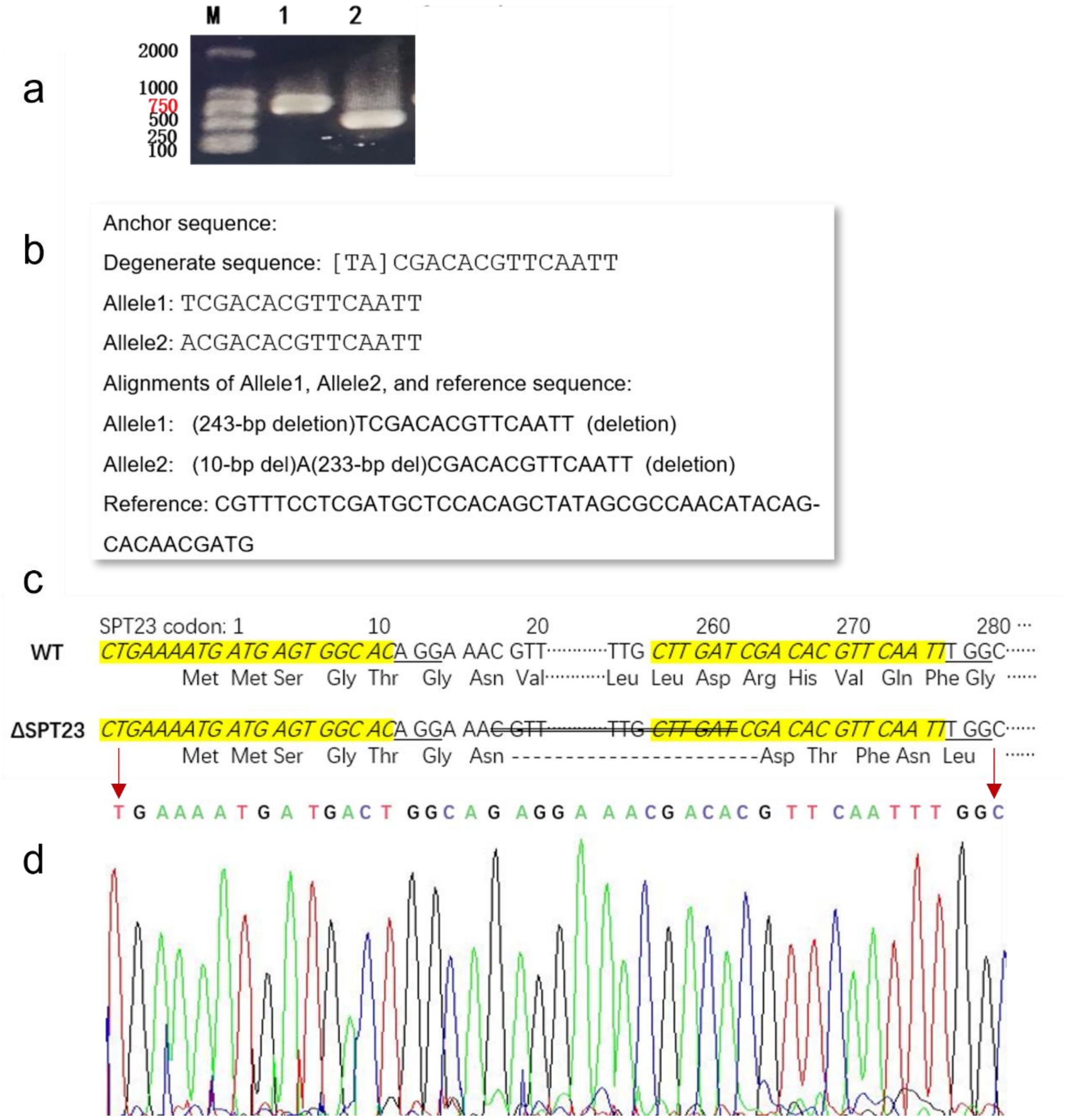

Fig. S2 Verification of *SPT23*Δ recombinant strain. **a**. PCR verification. M: DL2000 marker; L1: before gene editing; L2: after gene editing. **b**. Degenerated sequence analysis of PCR products using the DsDecodeM program. **c**. Schematic for the *spt23* frame shift deletion. **d**. Sanger sequencing confirmation of the *SPT23*Δ strain.

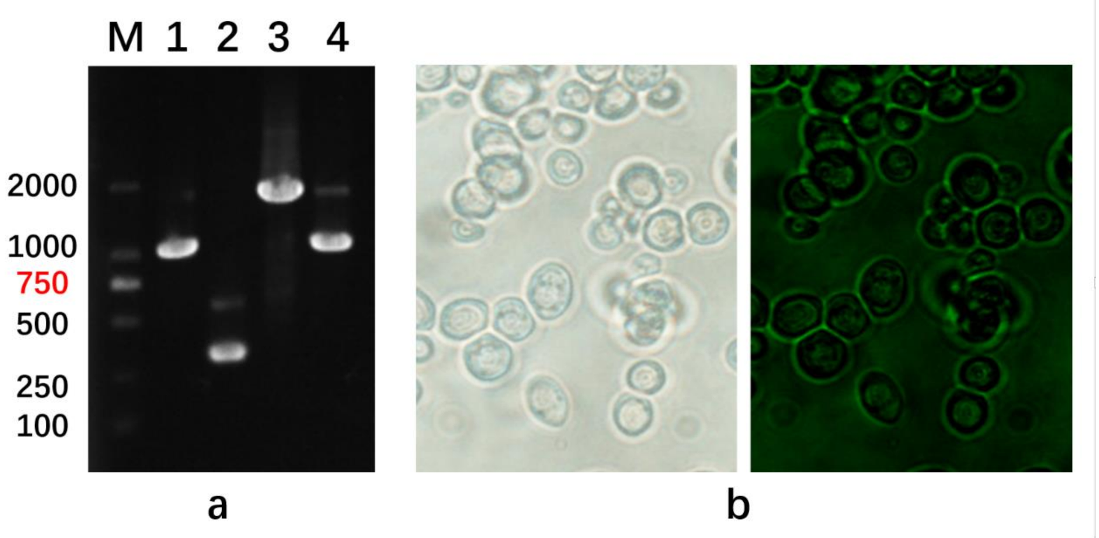

Fig. S3 Verification of *gfp-spt23* genomic integration. **a**. PCR verification. M: DL2000 marker; L1: *gfp* fragment; L2: partial *spt23* fragment; L3: *gfp-spt23* fusion fragment; L4: PCR verification of *gfp-spt23* genomic integration. **b**. Fluorescence microscopy examination of the *gfp-spt23* integration strain. Left image: Bright field microscopy image; Right image: green fluorescence field microscopy image.

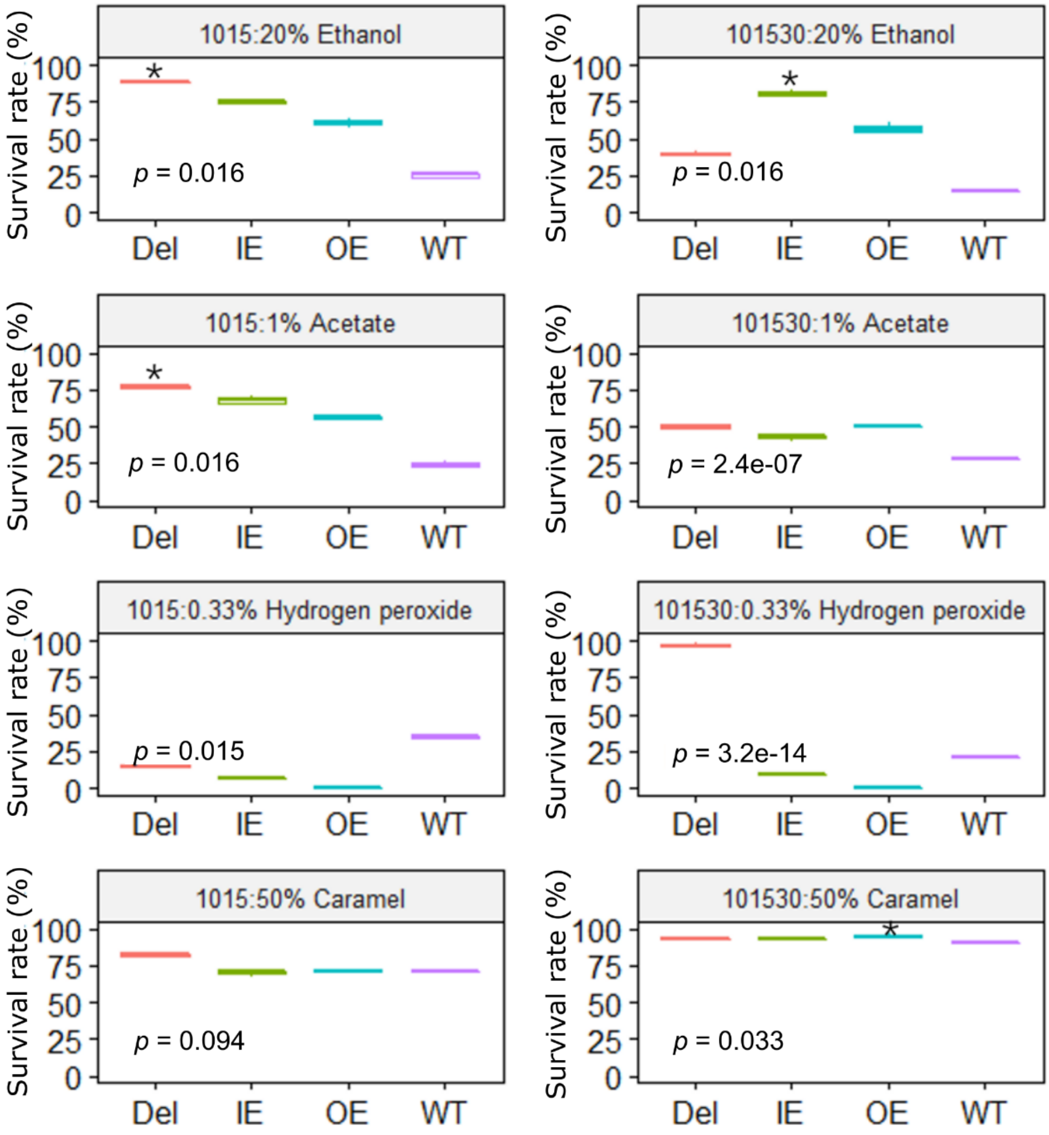

Fig. S4 Survival rates (%) under different environmental stresses. *Statistically significant difference compared to the WT strain ($p < 0.05$).

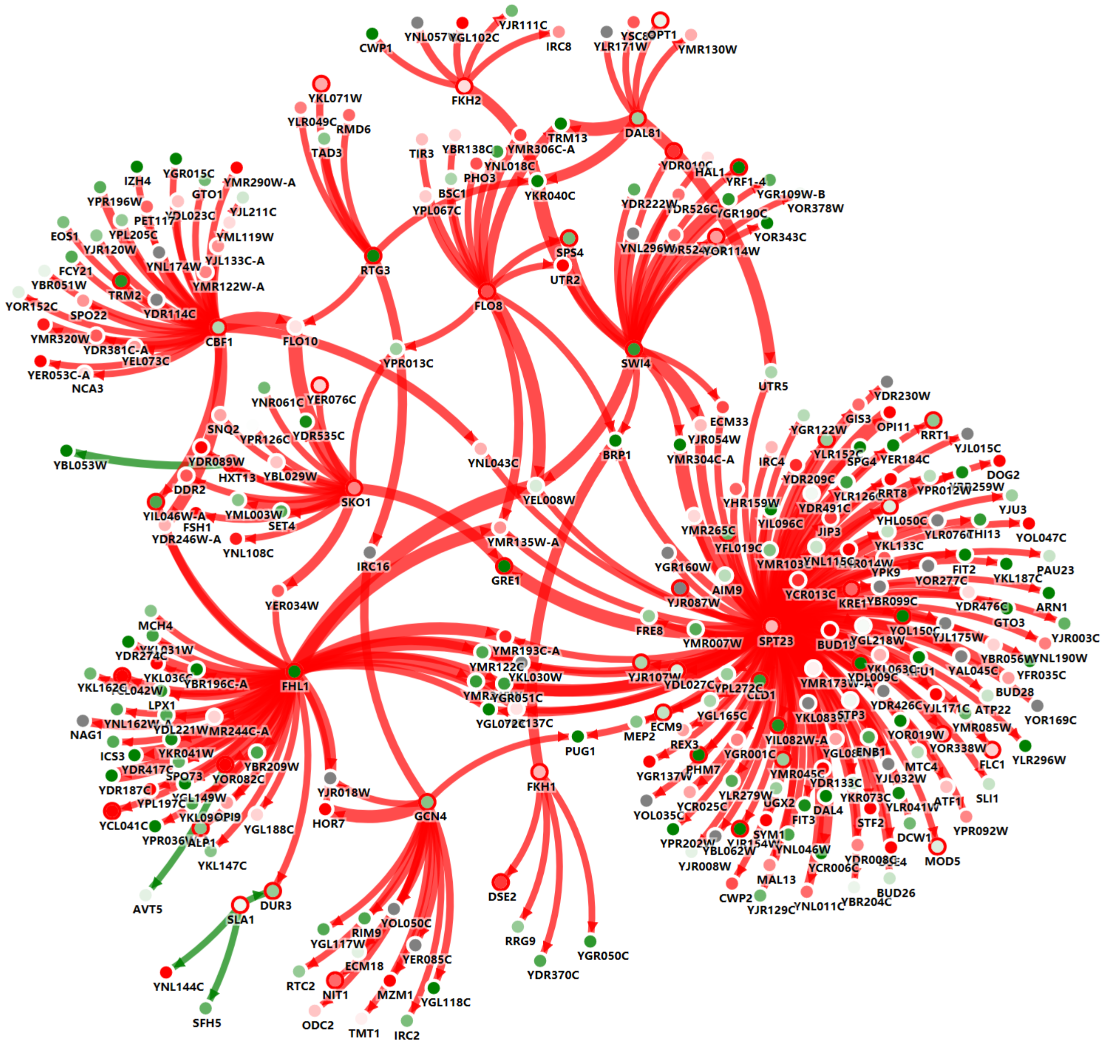

Fig. S5 Key drive mutant genes detected with the PheNetic software program when cultures were grown at 16 h.

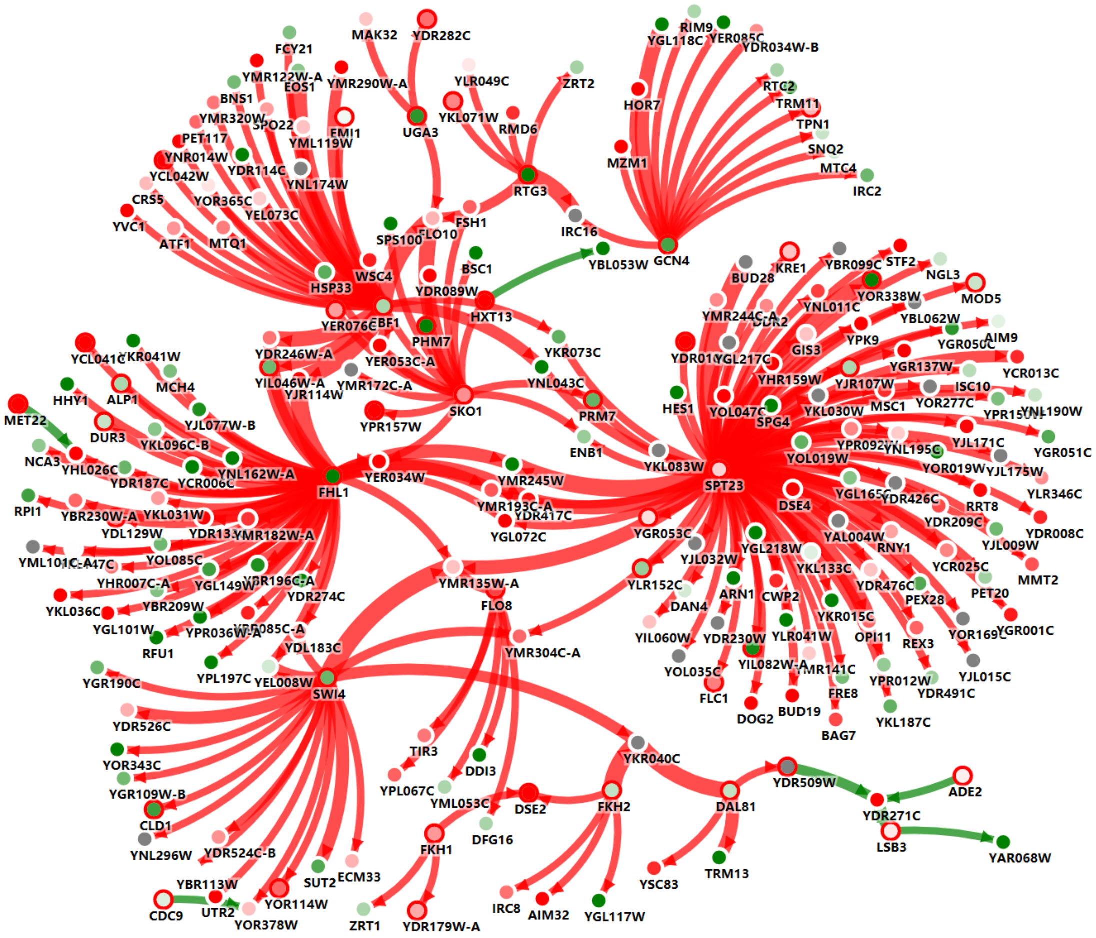

Fig. S6 Key drive mutant genes detected with the PheNetic software program when cultures were grown at 40 h.

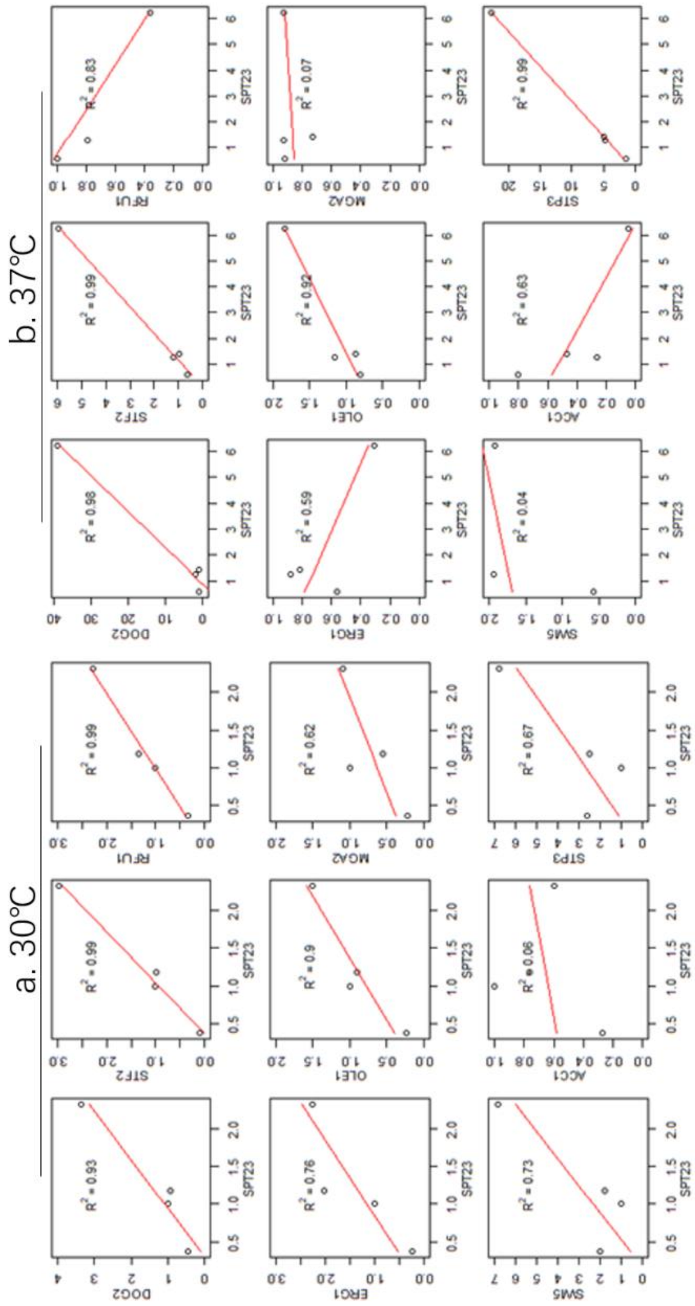

Fig. S7 Correlation of gene expression with *spt23* expression levels from cultures grown at (a) 30°C and (b) 37°C.

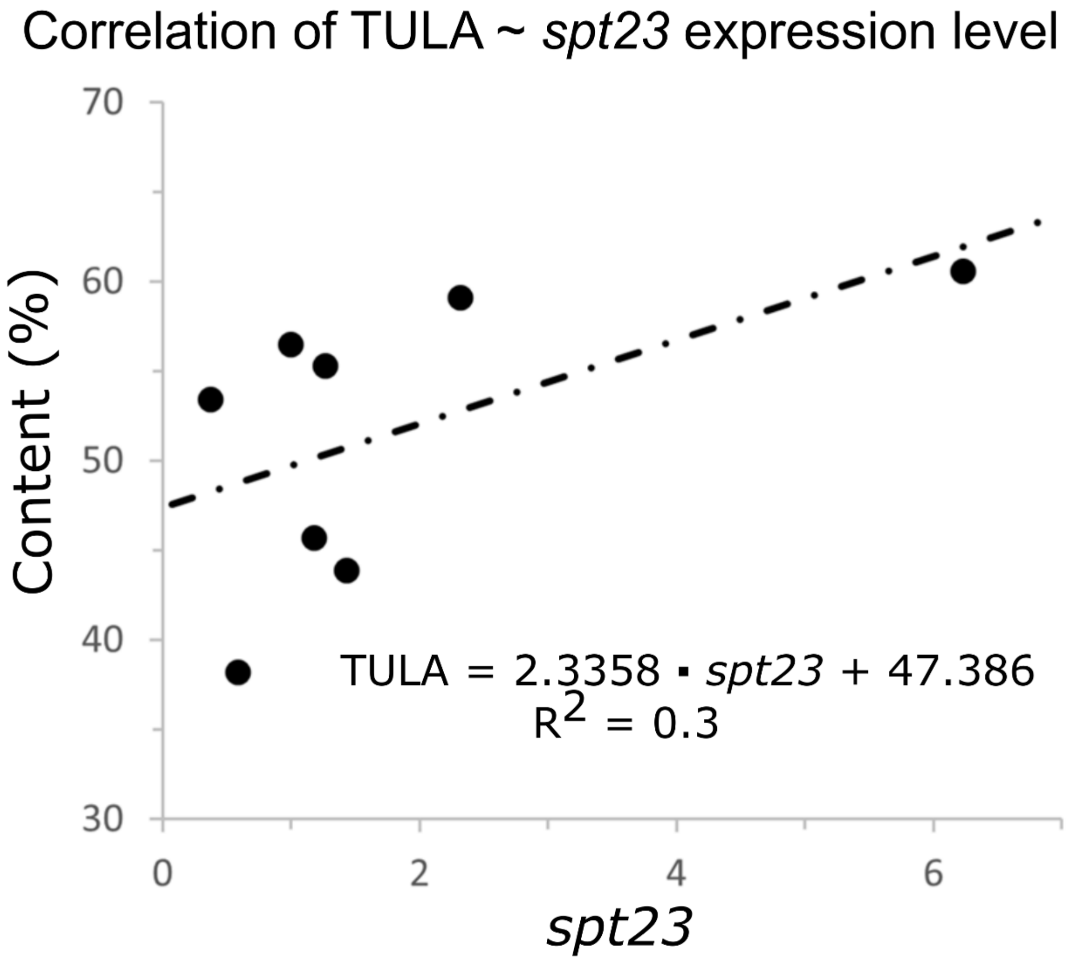

Fig. S8 Correlation between TULA contents and expression levels of *spt23*.

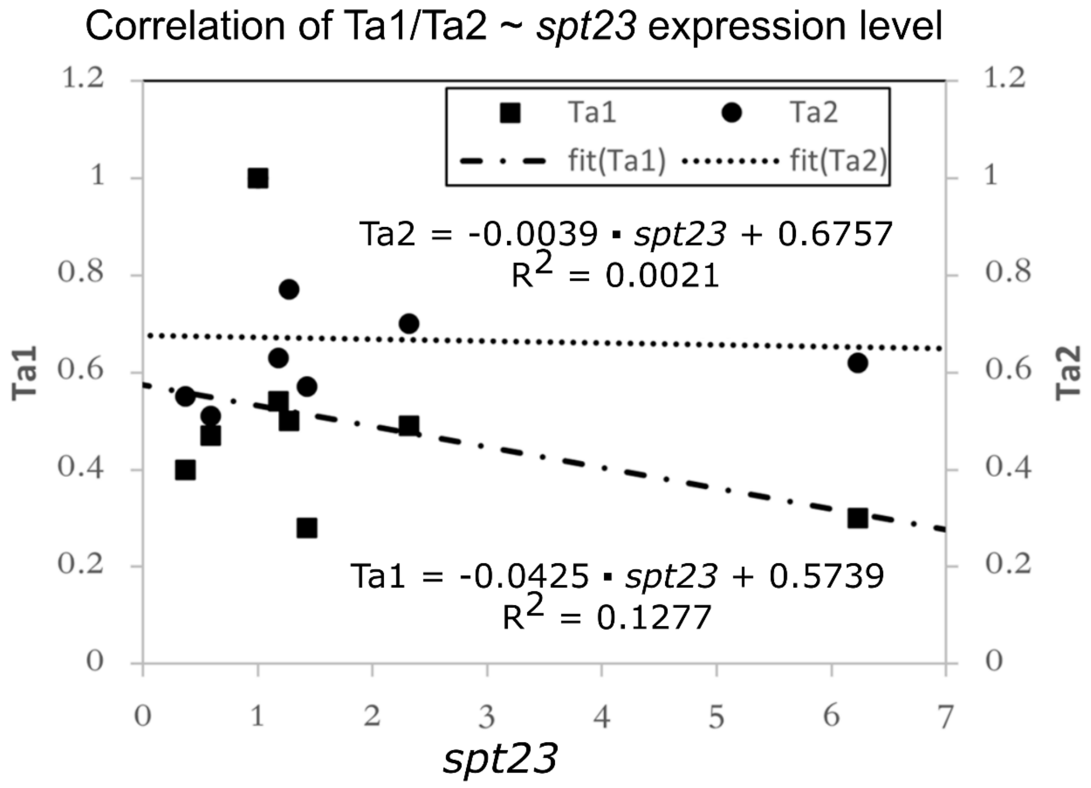

Fig. S9 Correlation between transposon activity (calculated as Ta1 and Ta2) and *spt23* expression levels.
